# A Randomized Study of the Effects of Additional Fruit and Nuts Consumption on Hepatic Fat Content, Cardiovascular Risk Factors and Basal Metabolic Rate

**DOI:** 10.1371/journal.pone.0147149

**Published:** 2016-01-20

**Authors:** Christian Agebratt, Edvin Ström, Thobias Romu, Olof Dahlqvist-Leinhard, Magnus Borga, Per Leandersson, Fredrik H. Nystrom

**Affiliations:** 1 Department of Medical and Health Sciences, Faculty of Medicine and Health Sciences, Linköping University, Linköping, Sweden; 2 Center for Medical Image Science and Visualization, Faculty of Medicine and Health Sciences, Linköping University, Linköping, Sweden; 3 Department of Clinical and Experimental Medicine, Faculty of Medicine and Health Sciences, Linköping University, Linköping, Sweden; 4 Department of Biomedical Engineering, Faculty of Medicine and Health Sciences, Linköping University, Linköping, Sweden; McMaster University, CANADA

## Abstract

**Background:**

Fruit has since long been advocated as a healthy source of many nutrients, however, the high content of sugars in fruit might be a concern.

**Objectives:**

To study effects of an increased fruit intake compared with similar amount of extra calories from nuts in humans.

**Methods:**

Thirty healthy non-obese participants were randomized to either supplement the diet with fruits or nuts, each at +7 kcal/kg bodyweight/day for two months. Major endpoints were change of hepatic fat content (HFC, by magnetic resonance imaging, MRI), basal metabolic rate (BMR, with indirect calorimetry) and cardiovascular risk markers.

**Results:**

Weight gain was numerically similar in both groups although only statistically significant in the group randomized to nuts (fruit: from 22.15±1.61 kg/m^2^ to 22.30±1.7 kg/m^2^, p = 0.24 nuts: from 22.54±2.26 kg/m^2^ to 22.73±2.28 kg/m^2^, p = 0.045). On the other hand BMR increased in the nut group only (p = 0.028). Only the nut group reported a net increase of calories (from 2519±721 kcal/day to 2763±595 kcal/day, p = 0.035) according to 3-day food registrations. Despite an almost three-fold reported increased fructose-intake in the fruit group (from 9.1±6.0 gram/day to 25.6±9.6 gram/day, p<0.0001, nuts: from 12.4±5.7 gram/day to 6.5±5.3 gram/day, p = 0.007) there was no change of HFC. The numerical increase in fasting insulin was statistical significant only in the fruit group (from 7.73±3.1 pmol/l to 8.81±2.9 pmol/l, p = 0.018, nuts: from 7.29±2.9 pmol/l to 8.62±3.0 pmol/l, p = 0.14). Levels of vitamin C increased in both groups while α-tocopherol/cholesterol-ratio increased only in the fruit group.

**Conclusions:**

Although BMR increased in the nut-group only this was not linked with differences in weight gain between groups which potentially could be explained by the lack of reported net caloric increase in the fruit group. In healthy non-obese individuals an increased fruit intake seems safe from cardiovascular risk perspective, including measurement of HFC by MRI.

**Trial Registration:**

ClinicalTrials.gov NCT02227511

## Background

There is a great interest in what to eat to avoid cardiovascular disease and obesity. Fruit has since long been advocated as a nutrient source which is rich in vitamins, antioxidants and fiber. The Swedish Food Agency for example, suggest and intake of 500 grams each day of fruit and vegetables in order to improve blood lipids and to reduce risk for cardiovascular disease, type 2 diabetes and cancer (http://www.livsmedelsverket.se/en/). However, when tested in randomized placebo controlled trials, the effects of added antioxidants or vitamins have so far generally not proven effective to reduce cardiovascular or other diseases [1]. A low-fat diet with increased intake of fruit and vegetables also had no effect on the primary endpoints, cardiovascular disease and cancer, in women in a large American prospective randomized trial [2]. In fact, in some studies there has been an increased prevalence of cardiovascular disease or cancer when vitamins were added to the diet [3, 4]. As explanation for these findings, some researchers suggest that purification of antioxidants or vitamins and hence the “artificial” administration in the form of pills, opposite to be a natural part of the diet, does not provide protection in a prudent way [5, 6]. Another concern with fruit has been the high content of sugar, in particular of fructose [7, 8]. The liver clears the fructose from the portal vein and a high intake of fructose can elevate hepatic fat content in humans [9, 10]. Indeed, a high level of hepatic, as in non-alcoholic fatty liver disease (NAFLD), is a hallmark of insulin resistance and is linked with increased risk for cardiovascular disease [11, 12].

Recently a diet with emphasis on extra intake of olive oil and nuts on top of a Mediterranean diet reduced incidence of cardiovascular disease when compared with the traditional low-fat diet based on carbohydrates from grain, corn, potatoes and rice [13]. In the traditional Mediterranean diet there are many food types that could be considered as healthful [14]. Nuts is one such food item that is rich in proteins and mono- and polyunsaturated fats that also contain fat-soluble vitamins, essential minerals, flavonoids and other micronutrients. Proteins and fat in nuts could hold an advantage when compared with the high carbohydrate content in fruit since those macronutrients might increase metabolic rate efficiently [14, 15], and this could potentially reduce future risk of obesity.

We performed a trial in healthy participants with a duration of two months. The participants were randomized to either supplement the diet with fruits or nuts, each at +7 kcal/kg bodyweight/day. This amount of energy corresponds closely to 500 grams fruit /day for a bodyweight of 70 kg (in reference to the Swedish Food Agency as above) assuming that the energy content in the fruit is 1 kcal/gram, which is typical of for example bananas. Serum levels of carotenoids and vitamins C and E were assessed to gain information about uptake of these micronutrients when the food composition was changed. Major endpoints were hepatic fat content, basal metabolic rate and cardiovascular risk markers.

## Materials and Methods

### Recruitment

The participants were recruited through local advertising at Linköping University. Exclusion criteria were significant pre-existing medical conditions or use of thyroid hormone replacement. Ongoing dietary supplementation, as in multivitamins were not allowed, but if the potential participant abstained from consuming the supplements two weeks prior to first blood sample of the trial, they could be enrolled.

### Blood sampling

Blood was drawn in the morning after a 10 hour over-night fast. Standard laboratory tests were analyzed according to routines at Department of Clinical Chemistry at the Linköping University Hospital. Plasma levels of major carotenoids (lutein+zeaxanthin, β-cryptoxanthin, lycopene, α- and β-carotene) and vitamin E were determined by high-performance liquid chromatography as described earlier [16]. To prevent oxidation, plasma samples used for analysis of vitamin C were mixed with 10% meta-phosphoric acid (1:1, *v/v*) before freezing. Vitamin C was analyzed with high-performance liquid chromatography as described previously [17], however, one modification was that a gradient elution starting with a mobile phase consisting of 50 mM KH_2_PO_4_, pH 3.2 and ending with 100% acetonitrile was used to obtain a more efficient washing of the column between injections. To reduce risk of errors, reference standard plasma was analyzed at each analytical run. Multiple analysis of this sample resulted in coefficients of variation within day and between days of 8.5% and 4.3%, respectively. To eliminate the risk of degradation, samples for analysis of carotenoids and vitamins were protected from direct sunlight. During the second round of blood samples, after 2 months, the female test subjects were booked 8 weeks +/- 1 day after the first collection of blood to counteract variations in hormone levels due to the menstrual cycle, the male participants thus filled the remaining available slots.

### Basal Metabolic Rate

Basal metabolic rate (BMR) was measured by indirect calorimetry (Quark RMR, Cosmed, Finland) and the equipment was calibrated daily. All participants rested lying down for 10 minutes before the BMR measurements were started and all measurement were performed within 4 days of the corresponding sampling of blood. Blood pressure in the left arm was recorded in the supine position with a standard 12 cm cuff after the BMR measurement. The average of two recordings from the mercury sphygmanometer was noted. The sagittal abdominal diameter was measured with a dedicated sliding beam in the supine position, after standardized exhalation [18].

### Nutritional intake

Diet registrations were performed at baseline and at the end of the trial periods during at least three consecutive days out of which one day was a Saturday or a Sunday. The participants were provided with dedicated scales (Soehnle 66100, Nassau, Germany) and note-books to weigh and record all food items that were consumed during these periods.

### Activity level

The activity level was measured with an accelerometer worn on the non-dominant wrist for three days (Actigraph wGT3X-BT, Actilife, Florida, USA) with licensed software Actilife (version 6.11.5). The measurement took place during the same time as the registration of food intake. Epoch-length, the time intervals for data collection, was set to 10 seconds. Scoring of the wear-time validated data was done using the algorithm Freedson Adult VM3 [19]. Non-wear time [19] and negative wear-sensor readings were excluded from the analyses.

### Quantification of liver steatosis and body composition

Magnetic Resonance Imaging was performed before and during the end of the intervention to assess the amount of fat in the liver in terms of proton density fat fraction (pdFF). The pdFF was measured based on three 23x23x30 mm^3^ regions of interests (ROIs) placed within the liver in the homogeneous liver parenchyma avoiding major blood vessels, bile ducts and the lateral margin of the liver. The ROI locations in the two examinations were matched. The operator was blinded to the intervention group of the subjects and in the acquisition order of the two acquisitions. The pdFF image series were computed offline from 6-echo spoiled gradient echo images [20] acquired on a Ingenia 3.0T (Philips Healthcare, Best, the Netherlands) with a flip angle of 3 degrees, repetition time of 6.4 ms, echo times of 1.15 + 0.81*n ms, water fat shift 0.18 pixels, matrix size 160x160 pixels, field of view 350x231x400 mm^3^ and 77 slices with a thickness of 6 mm. Multi-atlas segmentation was used for accurate automated measurements of abdominal fat [21] and thigh muscle volume [22].

### Intervention

Participants were randomized by drawing ballots to eat 7 kCal per kg bodyweight per day of either fruit or nuts. As an example, for a subject weighing 65 kg this corresponded to a daily intake of approximately 7 apples or 70 g of walnuts, respectively. There were no particular instructions given in what food to eat ahead of actual study start. The study lasted 8 weeks and the fruit or the nuts were recommended to be consumed between the regular meals. The participants bought and consumed the fruits or nuts and kept diaries of the amount they consumed throughout the study. They were consecutively reimbursed for the cost of the fruit or nuts. Apples and pears were particularly recommended since these are seasonal fruits in Sweden and representative for the country and the study took place during autumn of 2014 (from September to December). The fruit had to be fresh and not processed. The classifications of fruit and nuts were not made from a strict botanical categorization and small amounts of berries were allowed for the purpose of variation in the fruit group. Bananas were allowed as choice among “fruits” although they are classified as berries. Conversely, peanuts were considered an acceptable choice among nuts, although peanuts are classified as legumes.

### Ethics

The study was approved by the Regional Ethics Committee of Linköping (no 2014/170-31) and performed in accordance with the Declaration of Helsinki and it was registered at ClinicalTrials.gov with number NCT02227511. Written informed consent was obtained from all participating subjects

### Statistical analyses and power calculation

Statistical estimates were calculated using IBM SPSS Statistics 22 software (IBM Corporation, Somers, New York, USA). Comparisons within and between groups were done with Student’s paired and unpaired 2-tailed t-test for normally distributed data or as stated in the results section. Mean values and standard deviations are given. Linear correlations were done by using the Pearson product-moment method. Statistical significance refers to 2-sided p≤ 0.05. Levels of vitamin C that could not be detected were set to 1 μM in statistical calculations since the lower limit of detection for vitamin C was 2 μM.

Based on our earlier intervention studies of hepatic fat content [23, 24] the study had 80% power to detect a 50% change in hepatic fat content within either group.

## Results

**Fig 1:**
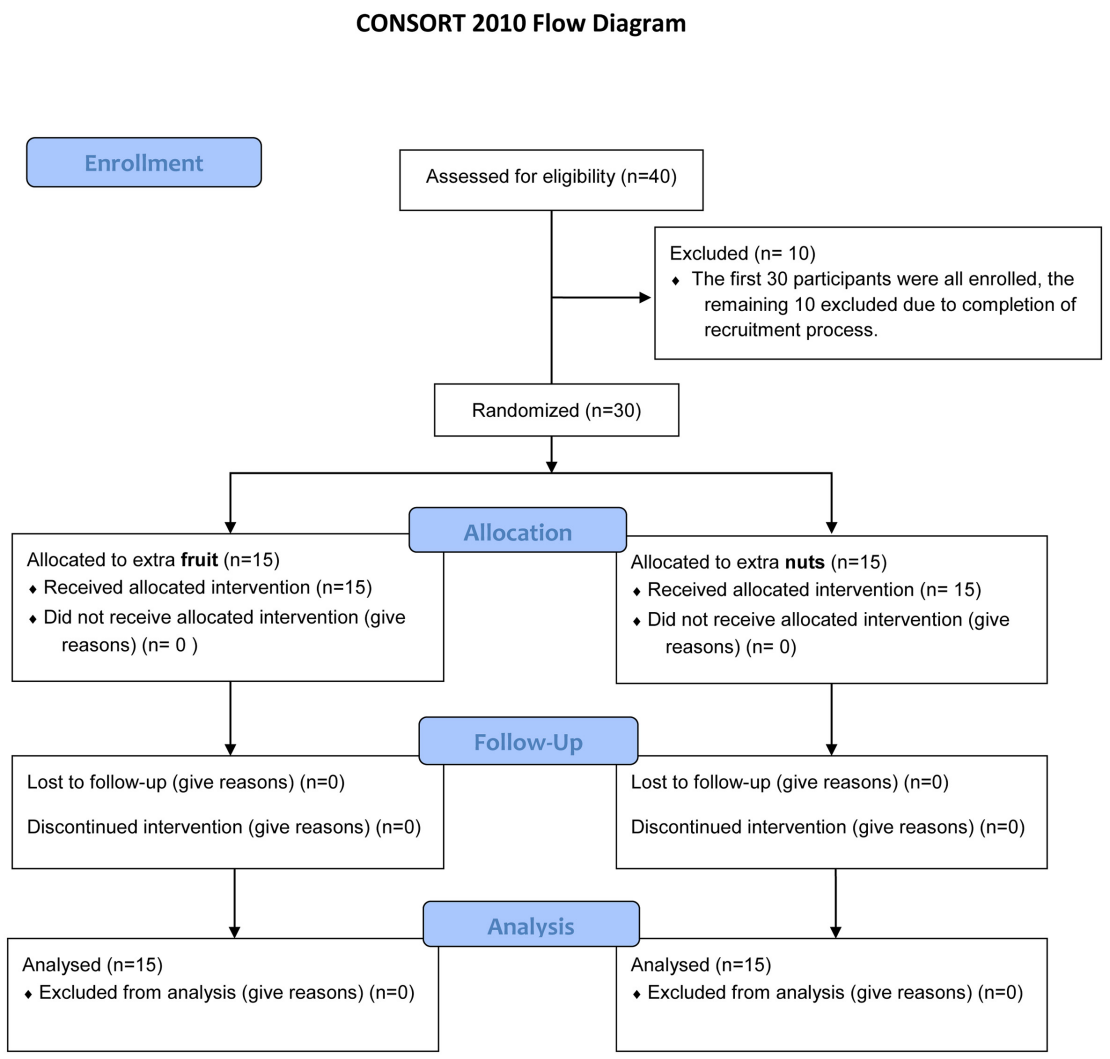
Flow chart of the study.

Forty potential participants responded to the advertising and the first 30 of these were enrolled in the study. All tested negative for hepatitis B and C and none had any exclusion criteria. The age of the final group of 18 males and 12 females (7 men and 8 women in the fruit group, 11 men and 4 women in the nut group, p = 0.15 for gender composition in the groups) was 23.5 ± 3.7 years and the corresponding BMI was 22.3 ±1.9 kg/m^2^. Twenty-nine of the thirty participants were students at Linköping University and one was employed by the University. There were no dropouts during the trial. Fig 1 shows flow-chart of the trial. Table 1 shows anthropometric data in the two groups at baseline and at the end of the study.

**Table 1:** Baseline data and effects of the intervention.

Variable	Group	Before	After	P within groups	P for change between groups
Weight (kg)	Fruit	66.45 ± 8.70	67.15 ± 9.04	0.13	0.95
	Nut	73.61 ± 9.01	74.28 ± 9.02	0.049	
BMI (kg/m^2^)	Fruit	22.15 ± 1.61	22.30 ± 1.7	0.24	0.83
	Nut	22.54 ± 2.26	22.73 ± 2.28	0.045	
SAD (cm)	Fruit	15.8 ± 1.2	15.8 ± 1.0	0.75	0.91
	Nut	16.3 ± 1.0	16.5 ± 0.78	0.49	
Metabolic rate	Fruit	1787 ± 278	1845 ± 240	0.26	0.52
(kcal/24h)	Nut	1931 ± 221	2031 ± 294	0.028	
Accelerometry	Fruit	942 ± 409	783 ± 237	0.071	0.046
(kcal/24h)	Nut	863 ± 228	926 ± 329	0.37	
Steps/day	Fruit	12672 ± 2640	11894 ± 2696	0.23	0.12
	Nut	10608 ± 2715	11107 ± 2545	0.34	
Energy intake	Fruit	2635 ± 933	2663 ± 773	0.9	0.37
(kcal/day)	Nut	2519 ± 721	2763 ± 595	0.035	
Fructose intake	Fruit	9.1 ± 6.0	25.6 ± 9.6	<0.0001	<0.0001
(gram/day)	Nut	12.4 ± 5.7	6.5 ± 5.3	0.007	
Systolic BP	Fruit	110.9 ± 7.7	104.2 ± 7.1	0.001	0.060
(mmHg)	Nut	113.5 ± 7.2	111.7 ± 6.9	0.35	
Diastolic BP	Fruit	67.6 ± 6.7	64.3 ± 8.0	0.11	0.48
(mmHg)	Nut	66.7 ± 7.0	64.9 ± 6.4	0.091	
Hepatic fat	Fruit	2.11 ± 0.75	2.21 ± 0.63	0.44	0.72
content (%)	Nut	2.09 ± 0.68	2.09 ± 0.59	0.99	
Abdominal subc.	Fruit	3.47 1.3	3.46 1.3	0.92	0.27
fat (l)	Nut	3.44 1.5	3.55 1.4	0.17	
Visceral fat	Fruit	1.05 0.38	1.00 0.41	0.17	0.16
volume (l)	Nut	1.16 0.42	1.19 0.45	0.50	
Thigh muscle	Fruit	10.1 2.2	10.2 2.16	0.46	0.87
volume (l)	Nut	11.4 2.3	11.5 2.2	0.32	
Triglycerides	Fruit	0.80 ± 0.39	0.84 ± 0.38	0.57	0.20
(mmol/l)	Nut	0.88 ± 0.36	0.80 ± 0.33	0.20	
Cholesterol	Fruit	4.44 ± 0.88	4.38 ± 0.91	0.49	0.46
(mmol/l)	Nut	4.09 ± 0.52	3.95 ± 0.45	0.09	
HDL chol.	Fruit	1.57 ± 0.36	1.55 ± 0.43	0.41	0.75
(mmol/l)	Nut	1.40 ± 0.26	1.39 ± 0.22	0.83	
LDL chol.	Fruit	2.54 ± 0.75	2.43 ± 0.69	0.13	0.85
(mmol/l)	Nut	2.33 ± 0.55	2.20 ± 0.42	0.11	
ApoA-1	Fruit	1.4 ±0 .23	1.43 ± 0.27	0.071	0.54
(mg/l)	Nut	1.33 ± 0.19	1.35 ± 0.19	0.57	
ApoB	Fruit	0.84 ± 0.17	0.83 ± 0.18	0.33	0.93
(mg/l)	Nut	0.77 ± 0.13	0.75 ± 0.10	0.44	
LDL/HDL-ratio	Fruit	1.62 ± 0.61	1.62 ± 0.46	0.99	0.40
	Nut	1.72 ± 0.50	1.62 ± 0.37	0.21	
ApoB/ApoA-1-	Fruit	0.61 ± 0.13	0.59 ± 0.13	0.041	0.77
ratio	Nut	0.59 ± 0.13	0.57 ± 0.01	0.33	
Sf-Insulin	Fruit	7.73 ± 3.1	8.81 ± 2.9	0.018	0.79
(pmol/l)	Nut	7.29 ± 2.9	8.62 ± 3.0	0.14	
HbA1c	Fruit	32.5 ± 2.4	31.8 ± 2.2	0.028	0.51
(mmol/mol)	Nut	31.9 ± 3.3	31.6 ± 3.7	0.68	
Sf-glucose	Fruit	5.09 ± 0.36	5.16 ± 0.19	0.40	0.45
(mmol/l)	Nut	5.26 ± 0.46	5.22 ± 0.29	0.73	

Abbreviations: SAD, sagittal abdominal diameter; Sf, serum fasting; Subc, subcutaneous.

Reported intake of fructose increased three-fold in the fruit group and was about halved in the nut group (p< 0.0001 for change between groups, Table 1). In the nut group the intake of energy from nuts increased 538 ± 142 kcal/day compared with baseline intake and the corresponding figure in the fruit group was 423 ± 96 kcal/day (p = 0.016 for comparison of the increase in the two groups). In the fruit group the reported intake of fruit during the intervention was 9.58 grams of fruit/day/kg bodyweight and the corresponding figure for the nut group was 1.24 grams of nuts/day/kg bodyweight. When the intakes from different nuts were compared and expressed as gram/day, the most common sources were from cashews (47.4%), peanuts (14.1%), walnuts (8.1%), almonds (8.0%), pistachios (7.8%), hazelnuts (5.8%), and Brazil nuts (3.5%) with less than 1% coming from macadamias or pecan nuts etc. A total of 3.4% of the intake of nuts was reported as unspecified “nut mixes”. The corresponding results of proportions of different fruits in the fruit group were bananas (38.7%), apples (19.4%) citrus fruits (14.8%), pears (8.2%), melons (3.9%), grapes (3.2%) mangos (3.0%), kiwis (2.2%), or persimmons (1.8%) with less than 1% each from pineapples or plums etc. The compliance expressed as reported intake of nuts or fruit in kcal/day/kg bodyweight during the study period compared with the goal of 7 kcal/day/kg bodyweight was 107.6 ± 8.8% in the nut group and 98.8 ± 9.4% in the fruit group (p = 0.014 between groups).

**Fig 2:**
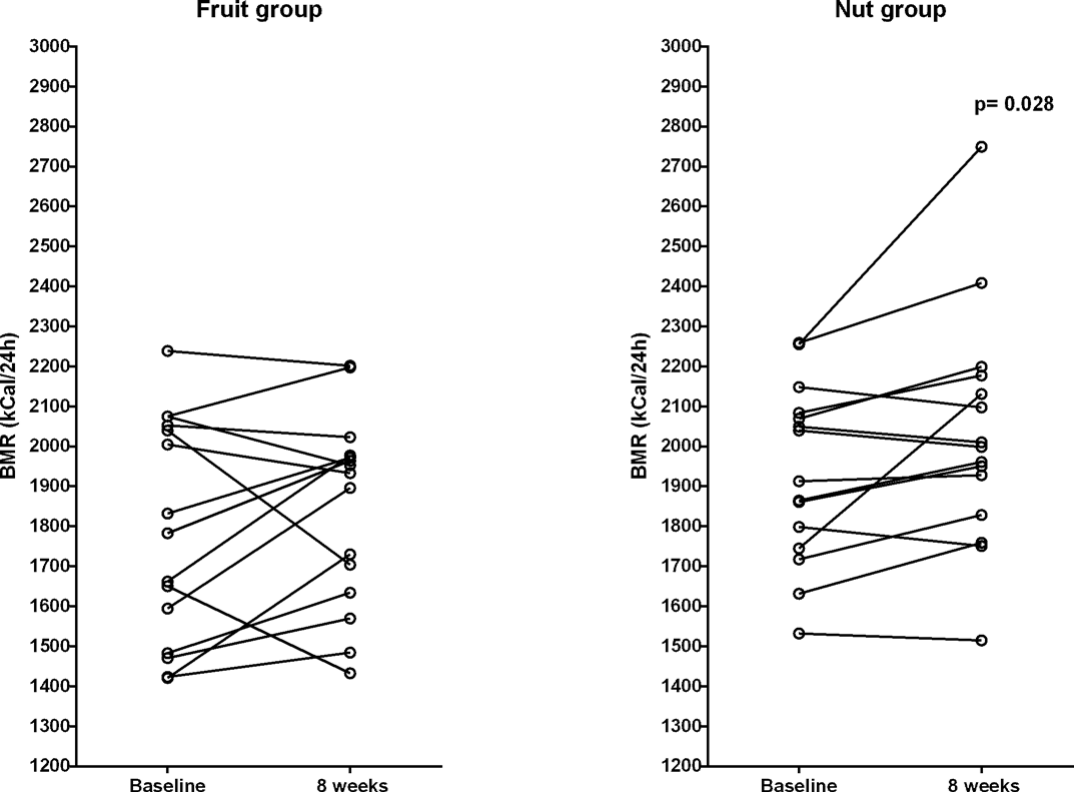
Individual changes of basal metabolic rate in the groups randomized to consume extra fruit or extra nuts. The basal metabolic rate measured by indirect calorimetry increased in the nut group only (fruit group: from 1787 ± 278 kcal/24h to 1845 ± 240 kcal/24h, p = 0.26, nut group: from 1931 ± 221 kcal/24h to 2031 ± 294 kcal/24h, p = 0.028).

Weight gain was numerically similar in both groups although only statistically significant in the group randomized to consuming extra nuts. Reported energy intake increased in the nut group only while general physical activity during the same time-period suggested differences in the behavior patterns between the two groups with respect to a trend to reduced activity in the fruit group and a trend to increase in the nut group (p = 0.046 for changes between groups). The basal metabolic rate measured by indirect calorimetry increased in the nut group only (p = 0.028, Fig 2). As seen in the figure one participant had a particularly large increase of BMR in the nut group. After removal of the BMR data corresponding to this single participant the numerical increase in the nut group bordered on statistical significance (increase 79.4 ± 14 kcal/24h, p = 0.055) in a *post hoc* analysis.

One participant declined a second run of magnetic resonance imaging for personal reasons. When the final cohort was analyzed separately for gender there was a linear correlation between reported intake of sugars (mono- and di-saccharides) and hepatic fat content at baseline in the women (r = 0.76, p = 0.007) but not in men (r = 0.04, p = 0.9). There was no change hepatic fat content in either group (Table 1). Levels of lipoprotein(a) (Lp(a)) tended to decrease in the nut group and to increase in the fruit group and the change in levels between groups was statistically significant (p = 0.047). The ApoB/ApoA-1 ratio improved in the fruit group only (p = 0.041).

There was an imbalance of serum vitamin C levels at baseline between groups (Table 2).

**Table 2:** Levels of vitamin C (μM) and the ratio vitamin E and carotenoids per cholesterol (μM/mM cholesterol) at start and after 8 weeks. Mean ± SD.

Variable	Group	Start	+ 8 weeks	P for change within group	P for change between groups
Vitamin C	Fruit	16.3 ± 11*	28.7 ± 15*	0.017	0.53
	Nut	8.7 ± 6.0*	17.2 ± 9.4*	0.009	
α-Tocopherol	Fruit	6.1 ± 0.71	6.65 ± 1.4	0.033	0.005
	Nut	6.6 ± 0.64	6.24 ± 0.71	0.067	
γ-tocopherol	Fruit	0.34 ± 0.14	0.35 ± 0.11	0.82	0.13
	Nut	0.37 ± 0.17	0.47 ± 0.15	0.081	
α-Carotene	Fruit	0.087 ± 0.048	0.078 ± 0.032	0.29	0.75
	Nut	0.076 ± 0.038	0.063 ± 0.047	0.083	
β-Carotene	Fruit	0.25 ± 0.10	0.23 ± 0.12	0.40	0.26
	Nut	0.25 ± 0.079	0.21 ± 0.059	<0.0001	
Lutein	Fruit	0.10 ± 0.031	0.10 ± 0.034	0.44	0.83
	Nut	0.096 ± 0.029	0.097 ± 0.038	0.82	
Lycopene	Fruit	0.18 ± 0.069	0.17 ± 0.076	0.69	0.52
	Nut	0.20 ± 0.068	0.18 ± 0.059	0.12	
Cryptoxanthin	Fruit	0.058 ± 0.043	0.068 ± 0.060	0.20	0.20
	Nut	0.038 ± 0.020	0.037 ± 0.015	0.74	

*The differences of vitamin C levels between groups at baseline (p = 0.023) and at the end (p = 0.017) of the trial were statistically significant.

This was at least partially a consequence of two participants who were randomized to the nut group and one randomized to the fruit group who had levels of vitamin C that could not be detected at study start, i.e. a level < 2μM. When vitamin C levels were re-measured in these three males, 3–6 months after finalization of the study, when they stated that they had resumed to their regular food patterns, the levels were normal as compared with the other remaining participants (levels of 18 μM, 46 μM and 64 μM, respectively).

Alpha tocopherol/cholesterol-ratio increased in the fruit group and decreased numerically in the nut group, the difference in changes between groups was statistically significant as seen in Table 2 (p = 0.005). The ratio β-carotene/cholesterol decreased in the nut group (p<0.0001) but for other carotenoids there were no significant changes in the carotenoid/cholesterol ratios.

## Discussion

According to self-reported intake of fructose, levels increased several-fold in the fruit group while decreasing in the nut group. In contrast to these findings, there was no change in hepatic fat content, nor was there an increase in serum triglycerides which has earlier been shown to accompany increased fructose intake [25]. Potential explanations for the unaffected liver fat content could be that the young and healthy participants in the trial, with high level of physical activity, have an inherent metabolic state in which the addition of fructose from fruit is not adverse for the liver. Another possibility is that the liver could adapt and improve its capacity to metabolize carbohydrates and fructose within the two month period that the trial lasted. We have earlier shown such adaptations in a 4-week study of considerably greater caloric intake than the trial presented herein [26]. The reason for choosing a time period of two months, however, was to gain more information about this possible adaptation after a longer time period.

It is of course possible that the data from the diet registrations were not entirely correct, as has been found in studies aimed to assess precision of diet registrations [27–30]. However, both groups did show the same numerical increase in body weight, + 0.7 kg, which supported a true net increase in caloric intake. In the nut group only, there was an increased basal metabolic rate (+ 100 kcal/24h). In the fruit group, on the other hand, the trend was in favor of lowered physical activity in such a way that the difference between changes in the groups was statistically significant in this respect. If these opposing trends of changes in physical activity were indeed consequences of the imposed change in diet with different amounts of fruit intake, our data do not support fruits as vice source of energy to increase level of physical activity [31]. However, given the small size of the study regarding variables with such limited reproducibility as accelerometry and diet registrations, this could have been a finding due to chance. Our study was powered based on potential changes in hepatic fat content by magnetic resonance imaging which has higher reproducibility.

The numerical increase in body weight in the participants randomized to fruit was smaller than what could be expected from the trial design since this group, as stated above, did not show an increase in basal metabolic rate and also there was a trend to decreased physical activity. In theory, the expected net increase in energy of 66.4 kg (mean weight in the fruit group) x 7 kcal/kg/day x 56 days should have amounted to + 26 000 kcal. Theoretically this would have caused a weight increase of 2.9 assuming that it would be stored as fat tissue (assuming 9000 kcal/kg fat tissue) and if the metabolic rate remained unchanged. However, the participants in the fruit group did not report an increase of caloric intake, so most likely the intake of other sources of food were diminished to match the extra intake of energy from fruit.

Although the levels of vitamin C did increase in both groups, which are in line with both an increase intake of nuts or of fruit, we were surprised that three participants had very low levels at baseline. It cannot be excluded that this was caused by technical problems, but we find this unlikely since the actual HPLC chromatograms for these three samples looked normal when scrutinized.

In a large American population survey (NHANES) during the years 2003–2004 [32] it was found that 7.1% had vitamin C deficiency when this was defined as a level < 11.4 μM. In this NHANES report the 5^th^ percentile of serum vitamin C in men 20–39 years old was 6.9 μM. In our group, the mean value at baseline was 12.5 ± 9.4 μM whereas mean levels in the total NHANES cohort was 51.4 μM. This difference was unexpected and as mentioned above not likely due to errors during the blood sampling procedure or handling, storage and analysis of the samples. The participants in our trial were mostly students at the medical faculty and one might speculate that their dietary habits are somewhat poorer than that among the general public. Compared to many other food products, vitamin- rich fruits and vegetables are costly and this could be one reason why participants in our study, without a regular income, were more prone to buy and consume less vitamin C-rich foods ahead of the study start.

None of our participants had any signs or symptoms of scurvy, as based on clinical investigations and queries that also included questions about dental status (not shown). During the study period there was an increase in vitamin C levels in both groups. This increase was however modest and the reason for that could be that nuts and also fruits like apples and pears, that were recommended, contain only small amounts of vitamin C.

The reduction of serum β-carotene in the nut group is in line with the reduced reported intake of fructose in such manner that this would reflect reduced intake of fruit. Nuts in general only contain minute amounts of β-carotene (and other carotenoids). The small but significant increase in α-tocopherol among participants in the fruit group was somewhat unexpected since tocopherols are more associated to consumption of nuts and seeds [33] than to fruits. However, to some extent tocopherols can also be found in fruits and we found that the fruit group, but not nut group, significantly increased the α-tocopherol per mmol cholesterol-ratio.

Fasting insulin increased numerically in both groups which would be expected based on the study design aiming for increased energy consumption and body weight. The increase was statistically significant only in the fruit group, however, suggesting that the increase in carbohydrates, sugars, affected insulin sensitivity in a more specific manner in this group. The high reproducibility of the HbA1c analysis made it possible to discern a small statistically significant lowering of 0.7 mmol/mol in the fruit group, the clinical relevance of which is doubtful. The most likely explanation for this seemingly contradictory finding, increased intake of sugars but reduced mean glucose, would be that the HbA1c method specifically measures glucosilation of the hemoglobin molecule, and hence that it does not detect “fructosilation”. Thus this implies that previously ingested glucose was replaced by fructose in the diet in this group.

In general blood lipids showed small numerical changes in the trial. However, serum Lp(a) changed significantly between groups in a manner that would suggest increased nut intake to be more advantageous than fruit. This could be of some interest since levels of Lp(a) are not affected much by conventional treatment of hypercholesterolemia, including use of statins and ezetimibe [34].

Limitations of the study include the duration of two months and that we only included healthy non-obese individuals. These short-comings were consequences of the trial design with a power calculation that was based on the assumption that no participants had overt liver steatosis at baseline. The statistical power to test effects on hepatic fat content was of reasonable strength, 80%, but the lower reproducibility of many other variables that we studied yielded more limited power to detect potentially small changes induced by the interventions. It could also be argued that it was a draw-back that no specific kind of nuts or fruit were advocated, or even provided, to the participants. However, the reason for this design was to make it more clinically relevant and to more closely relate to recommendations by for example the Swedish Food Agency as to have a large intake of fruit intake in general, and hence the agency does not recommend any specific fruit. We also acknowledge that the limited size of the study precluded use of multivariate analyses.

In summary we found small effects on cardiovascular risk factors by comparing extra intake of nuts with extra intake of fruit. Although basal metabolic rate increased in the nut-group only this was not linked with differences in weight gain between groups which potentially could be explained by the lack of caloric increase in the fruit group. In healthy non-obese individuals a large increase of fruit intake seems safe from cardiovascular risk perspective, including measurement of hepatic fat content with MRI-based technique. It is quite possible, however, that other results would have been found had we recruited obese and insulin-resistant participants.

## Supporting Information

S1 File CONSORT checklist. (DOC)

S2 File Translated research protocol. (DOCX)

S3 File Research protocol (Swedish). (DOC)

S4 File Translated ethical application form. (DOCX)

S5 File Ethical application form (Swedish). (DOC)

## References

[1] Bjelakovic G, Nikolova D, Gluud LL, Simonetti RG, Gluud C. Mortality in randomized trials of antioxidant supplements for primary and secondary prevention: systematic review and meta-analysis. JAMA: the journal of the American Medical Association. 2007;297(8):842–57. 10.1001/jama.297.8.842 . 17327526

[2] Howard BV, Van Horn L, Hsia J, Manson JE, Stefanick ML, Wassertheil-Smoller S, et al Low-fat dietary pattern and risk of cardiovascular disease: the Women's Health Initiative Randomized Controlled Dietary Modification Trial. JAMA: the journal of the American Medical Association. 2006;295(6):655–66. . 16467234 10.1001/jama.295.6.655

[3] Ebbing M, Bonaa KH, Nygard O, Arnesen E, Ueland PM, Nordrehaug JE, et al Cancer incidence and mortality after treatment with folic acid and vitamin B12. JAMA: the journal of the American Medical Association. 2009;302(19):2119–26. 10.1001/jama.2009.1622 19920236

[4] Lonn E, Bosch J, Yusuf S, Sheridan P, Pogue J, Arnold JM, et al Effects of long-term vitamin E supplementation on cardiovascular events and cancer: a randomized controlled trial. JAMA: the journal of the American Medical Association. 2005;293(11):1338–47. Epub 2005/03/17. doi: 293/11/1338 [pii] 10.1001/jama.293.11.1338 . 15769967

[5] Lazzeroni M, Gandini S, Puntoni M, Bonanni B, Gennari A, DeCensi A. The science behind vitamins and natural compounds for breast cancer prevention. Getting the most prevention out of it. Breast. 2011;20 Suppl 3:S36–41. 10.1016/S0960-9776(11)70292-2 . 22015291

[6] Edefonti V, Hashibe M, Parpinel M, Turati F, Serraino D, Matsuo K, et al Natural vitamin C intake and the risk of head and neck cancer: A pooled analysis in the International Head and Neck Cancer Epidemiology Consortium. Int J Cancer. 2015;137(2):448–62. 10.1002/ijc.29388 25627906 PMC4428957

[7] Tappy L, Le KA, Tran C, Paquot N. Fructose and metabolic diseases: new findings, new questions. Nutrition. 2010;26(11–12):1044–9. 10.1016/j.nut.2010.02.014 . 20471804

[8] Moeller SM, Fryhofer SA, Osbahr AJ 3rd, Robinowitz CB, Council on S, Public Health AMA. The effects of high fructose syrup. Journal of the American College of Nutrition. 2009;28(6):619–26. . 20516261 10.1080/07315724.2009.10719794

[9] Lecoultre V, Egli L, Carrel G, Theytaz F, Kreis R, Schneiter P, et al Effects of fructose and glucose overfeeding on hepatic insulin sensitivity and intrahepatic lipids in healthy humans. Obesity. 2013;21(4):782–5. 10.1002/oby.20377 . 23512506

[10] Maersk M, Belza A, Stodkilde-Jorgensen H, Ringgaard S, Chabanova E, Thomsen H, et al Sucrose-sweetened beverages increase fat storage in the liver, muscle, and visceral fat depot: a 6-mo randomized intervention study. The American journal of clinical nutrition. 2012;95(2):283–9. Epub 2011/12/30. doi: ajcn.111.022533 [pii] 10.3945/ajcn.111.022533 . 22205311

[11] Moore JB. Non-alcoholic fatty liver disease: the hepatic consequence of obesity and the metabolic syndrome. The Proceedings of the Nutrition Society. 2010;69(2):211–20. 10.1017/S0029665110000030 . 20158939

[12] Kim NH, Park J, Kim SH, Kim YH, Kim DH, Cho GY, et al Non-alcoholic fatty liver disease, metabolic syndrome and subclinical cardiovascular changes in the general population. Heart. 2014;100(12):938–43. 10.1136/heartjnl-2013-305099 . 24721975

[13] Estruch R, Ros E, Salas-Salvado J, Covas MI, Corella D, Aros F, et al Primary prevention of cardiovascular disease with a Mediterranean diet. The New England journal of medicine. 2013;368(14):1279–90. Epub 2013/02/26. 10.1056/NEJMoa1200303 . 23432189

[14] Claesson AL, Holm G, Ernersson A, Lindstrom T, Nystrom FH. Two weeks of overfeeding with candy, but not peanuts, increases insulin levels and body weight. Scand J Clin Lab Invest. 2009;69(5):598–605. 10.1080/00365510902912754 19396658

[15] Duarte Moreira Alves R, Boroni Moreira AP, Silva Macedo V, Brunoro Costa NM, Goncalves Alfenas Rde C, Bressan J. High-oleic peanuts increase diet-induced thermogenesis in overweight and obese men. Nutr Hosp. 2014;29(5):1024–32. 10.3305/nh.2014.29.5.7235 . 24951981

[16] Lidebjer C, Leanderson P, Ernerudh J, Jonasson L. Low plasma levels of oxygenated carotenoids in patients with coronary artery disease. Nutrition, metabolism, and cardiovascular diseases: NMCD. 2007;17(6):448–56. 10.1016/j.numecd.2006.02.006 . 17134954

[17] Hagfors L, Leanderson P, Skoldstam L, Andersson J, Johansson G. Antioxidant intake, plasma antioxidants and oxidative stress in a randomized, controlled, parallel, Mediterranean dietary intervention study on patients with rheumatoid arthritis. Nutr J. 2003;2:5 10.1186/1475-2891-2-5 12952549 PMC194256

[18] Dahlen EM, Bjarnegard N, Lanne T, Nystrom FH, Ostgren CJ. Sagittal abdominal diameter is a more independent measure compared with waist circumference to predict arterial stiffness in subjects with type 2 diabetes—a prospective observational cohort study. Cardiovascular diabetology. 2013;12:55 Epub 2013/03/30. 10.1186/1475-2840-12-55 1475-2840-12-55 [pii]. 23536999 PMC3637516

[19] Choi L, Liu Z, Matthews CE, Buchowski MS. Validation of accelerometer wear and nonwear time classification algorithm. Medicine and science in sports and exercise. 2011;43(2):357–64. 10.1249/MSS.0b013e3181ed61a3 20581716 PMC3184184

[20] Yu H, Shimakawa A, McKenzie CA, Brodsky E, Brittain JH, Reeder SB. Multiecho water-fat separation and simultaneous R2* estimation with multifrequency fat spectrum modeling. Magnetic resonance in medicine: official journal of the Society of Magnetic Resonance in Medicine / Society of Magnetic Resonance in Medicine. 2008;60(5):1122–34. 10.1002/mrm.21737 18956464 PMC3070175

[21] Leinhard OD, Johansson A, Rydell J, Smedby O, Nystrom F, Lundberg P, et al., editors. Quantitative Abdominal Fat Estimation Using MRI. 19th International Conference on Pattern Recognition; 2008; Tampa, FL.

[22] Karlsson A, Rosander J, Romu T, Tallberg J, Gronqvist A, Borga M, et al Automatic and quantitative assessment of regional muscle volume by multi-atlas segmentation using whole-body water-fat MRI. Journal of magnetic resonance imaging: JMRI. 2015;41(6):1558–69. 10.1002/jmri.24726 . 25111561

[23] Kechagias S, Ernersson A, Dahlqvist O, Lundberg P, Lindstrom T, Nystrom FH. Fast-food-based hyper-alimentation can induce rapid and profound elevation of serum alanine aminotransferase in healthy subjects. Gut. 2008;57(5):649–54. 10.1136/gut.2007.131797 18276725 PMC2565580

[24] Kechagias S, Zanjani S, Gjellan S, Leinhard OD, Kihlberg J, Smedby O, et al Effects of moderate red wine consumption on liver fat and blood lipids: a prospective randomized study. Annals of medicine. 2011;43(7):545–54. Epub 2011/05/24. 10.3109/07853890.2011.588246 . 21599573

[25] Angelopoulos TJ, Lowndes J, Zukley L, Melanson KJ, Nguyen V, Huffman A, et al The effect of high-fructose corn syrup consumption on triglycerides and uric acid. The Journal of nutrition. 2009;139(6):1242S–5S. 10.3945/jn.108.098194 . 19403709

[26] Kechagias S, Ernersson A, Dahlqvist O, Lundberg P, Lindstrom T, Nystrom FH. Fast food based hyper-alimentation can induce rapid and profound elevation of serum alanine aminotransferase in healthy subjects. Gut. 2008 . 18276725 10.1136/gut.2007.131797 PMC2565580

[27] Emond JA, Patterson RE, Jardack PM, Arab L. Using doubly labeled water to validate associations between sugar-sweetened beverage intake and body mass among White and African-American adults. International journal of obesity. 2014;38(4):603–9. 10.1038/ijo.2013.130 23867782 PMC3872257

[28] Lof M, Forsum E. Validation of energy intake by dietary recall against different methods to assess energy expenditure. J Hum Nutr Diet. 2004;17(5):471–80. . 15357701 10.1111/j.1365-277X.2004.00554.x

[29] Stice E, Durant S. Elevated objectively measured but not self-reported energy intake predicts future weight gain in adolescents. Appetite. 2014;81:84–8. 10.1016/j.appet.2014.06.012 24930597 PMC4128488

[30] Wallhuss A, Isik M, Nystrom FH. Comparison of the subjective sense of high or low metabolism and objectively measured resting metabolic rate. Scand J Clin Lab Invest. 2010;70(5):334–7. Epub 2010/06/01. 10.3109/00365513.2010.491125 . 20509821

[31] Lamprecht M. Supplementation with mixed fruit and vegetable concentrates in relation to athlete's health and performance: scientific insight and practical relevance. Med Sport Sci. 2012;59:70–85. 10.1159/000341960 . 23075557

[32] Schleicher RL, Carroll MD, Ford ES, Lacher DA. Serum vitamin C and the prevalence of vitamin C deficiency in the United States: 2003–2004 National Health and Nutrition Examination Survey (NHANES). The American journal of clinical nutrition. 2009;90(5):1252–63. 10.3945/ajcn.2008.27016 . 19675106

[33] Souza RG, Gomes AC, Naves MM, Mota JF. Nuts and legume seeds for cardiovascular risk reduction: scientific evidence and mechanisms of action. Nutr Rev. 2015;73(6):335–47. 10.1093/nutrit/nuu008 . 26011909

[34] Keller C. Apheresis in coronary heart disease with elevated Lp (a): a review of Lp (a) as a risk factor and its management. Ther Apher Dial. 2007;11(1):2–8. 10.1111/j.1744-9987.2007.00449.x . 17309568

