# Correction: A Randomized Study of the Effects of Additional Fruit and Nuts Consumption on Hepatic Fat Content, Cardiovascular Risk Factors and Basal Metabolic Rate

**DOI:** 10.1371/journal.pone.0209450

**Published:** 2018-12-13

**Authors:** Christian Agebratt, Edvin Ström, Thobias Romu, Olof Dahlqvist-Leinhard, Magnus Borga, Per Leandersson, Fredrik H. Nystrom

There is an error in the penultimate sentence under the subheading “Results” in the Abstract section. The correct sentence is: The numerical increase in fasting insulin was statistically significant only in the fruit group (from 7.73±3.1 mIE/L to 8.81±2.9 mIE/L, p = 0.018, nuts: from 7.29±2.9 mIE/L to 8.62±3.0 mIE/L, p = 0.14).

In Table 1, the Variable “Sf-Insulin” uses the unit pmol/L. The correct unit is mIE/L. Please see the corrected Table 1 here.

**Table 1:** Baseline data and effects of the intervention.

Variable	Group	Before	After	P within groups	P for change between groups
Weight (kg)	Fruit	66.45 ± 8.70	67.15 ± 9.04	0.13	0.95
	Nut	73.61 ± 9.01	74.28 ± 9.02	0.049	
BMI (kg/m^2^)	Fruit	22.15 ± 1.61	22.30 ± 1.7	0.24	0.83
	Nut	22.54 ± 2.26	22.73 ± 2.28	0.045	
SAD (cm)	Fruit	15.8 ± 1.2	15.8 ± 1.0	0.75	0.91
	Nut	16.3 ± 1.0	16.5 ± 0.78	0.49	
Metabolic rate (kcal/24h)	Fruit	1787 ± 278	1845 ± 240	0.26	0.52
	Nut	1931 ± 221	2031 ± 294	0.028	
Accelerometry (kcal/24h)	Fruit	942 ± 409	783 ± 237	0.071	0.046
	Nut	863 ± 228	926 ± 329	0.37	
Steps/day	Fruit	12672 ± 2640	11894 ± 2696	0.23	0.12
	Nut	10608 ± 2715	11107 ± 2545	0.34	
Energy intake (kcal/day)	Fruit	2635 ± 933	2663 ± 773	0.9	0.37
	Nut	2519 ± 721	2763 ± 595	0.035	
Fructose intake (gram/day)	Fruit	9.1 ± 6.0	25.6 ± 9.6	<0.0001	<0.0001
	Nut	12.4 ± 5.7	6.5 ± 5.3	0.007	
Systolic BP (mmHg)	Fruit	110.9 ± 7.7	104.2 ± 7.1	0.001	0.060
	Nut	113.5 ± 7.2	111.7 ± 6.9	0.35	
Diastolic BP (mmHg)	Fruit	67.6 ± 6.7	64.3 ± 8.0	0.11	0.48
	Nut	66.7 ± 7.0	64.9 ± 6.4	0.091	
Hepatic fat content (%)	Fruit	2.11 ± 0.75	2.21 ± 0.63	0.44	0.72
	Nut	2.09 ± 0.68	2.09 ± 0.59	0.99	
Abdominal subc. fat (l)	Fruit	3.47 1.3	3.46 1.3	0.92	0.27
	Nut	3.44 1.5	3.55 1.4	0.17	
Visceral fat volume (l)	Fruit	1.05 0.38	1.00 0.41	0.17	0.16
	Nut	1.16 0.42	1.19 0.45	0.50	
Thigh muscle volume (l)	Fruit	10.1 2.2	10.2 2.16	0.46	0.87
	Nut	11.4 2.3	11.5 2.2	0.32	
Triglycerides (mmol/l)	Fruit	0.80 ± 0.39	0.84 ± 0.38	0.57	0.20
	Nut	0.88 ± 0.36	0.80 ± 0.33	0.20	
Cholesterol (mmol/l)	Fruit	4.44 ± 0.88	4.38 ± 0.91	0.49	0.46
	Nut	4.09 ± 0.52	3.95 ± 0.45	0.09	
HDL chol. (mmol/l)	Fruit	1.57 ± 0.36	1.55 ± 0.43	0.41	0.75
	Nut	1.40 ± 0.26	1.39 ± 0.22	0.83	
LDL chol. (mmol/l)	Fruit	2.54 ± 0.75	2.43 ± 0.69	0.13	0.85
	Nut	2.33 ± 0.55	2.20 ± 0.42	0.11	
ApoA-1 (mg/l)	Fruit	1.4 ± 0.23	1.43 ± 0.27	0.071	0.54
	Nut	1.33 ± 0.19	1.35 ± 0.19	0.57	
ApoB (mg/l)	Fruit	0.84 ± 0.17	0.83 ± 0.18	0.33	0.93
	Nut	0.77 ± 0.13	0.75 ± 0.10	0.44	
LDL/HDL-ratio	Fruit	1.62 ± 0.61	1.62 ± 0.46	0.99	0.40
	Nut	1.72 ± 0.50	1.62 ± 0.37	0.21	
ApoB/ApoA-1 -ratio	Fruit	0.61 ± 0.13	0.59 ± 0.13	0.041	0.77
	Nut	0.59 ± 0.13	0.57 ± 0.01	0.33	
Sf-Insulin (mIE/l)	Fruit	7.73 ± 3.1	8.81 ± 2.9	0.018	0.79
	Nut	7.29 ± 2.9	8.62 ± 3.0	0.14	
HbA1c (mmol/mol)	Fruit	32.5 ± 2.4	31.8 ± 2.2	0.028	0.51
	Nut	31.9 ± 3.3	31.6 ± 3.7	0.68	
Sf-glucose (mmol/l)	Fruit	5.09 ± 0.36	5.16 ± 0.19	0.40	0.45
	Nut	5.26 ± 0.46	5.22 ± 0.29	0.73	
