# Supplementary material for: A Randomized Study of the Effects of Additional Fruit and Nuts Consumption on Hepatic Fat Content, Cardiovascular Risk Factors and Basal Metabolic Rate
Source: PLoS One. 2016 Jan 20;11(1):e0147149. doi: 10.1371/journal.pone.0147149 (PMC4720287; doi:10.1371/journal.pone.0147149)
Supplement: S2 File — (DOCX) [file pone.0147149.s002.docx]

**Research program:** **Does extra intake of fruit lead to less advantageous metabolic effects than nuts?**

**Background**

Many people eat snacks in between regular meals to reduce hunger. This often advocated by dieticians according to guidelines such as those by the Swedish Food Agency. It is often suggested that fruit is a good alternative for such snacking due to high content of vitamins. There are many companies that supply work places with fruit baskets that are paid by employers so that employees can eat one fruit a day. New data suggest that intake of sugars should be limited to reduce the obesity epidemic, but this debate mainly concerns added sugars in food. Fruit juices are still by some people considered to be healthy foods while others question all forms of sweet drinks and state that such drinks are harmful and increase risk for obesity. Many modern studies suggest that fructose, often found in fruit, is linked with increased risk for metabolic side effects including reduced insulin sensitivity to glucose uptake in the insulin-responsive cells, i.e. so called insulin resistance and also hepatic steatosis. So far, the interest to study effects of whole fruits, rather than fruit juice, has been very limited, in these respects. It could be argued that the fiber content in fruit that is chewed as whole fruit could constitute a protection against the metabolic risks of such intake, but according to our knowledge this has not been tested in humans in a randomized manner in which hepatic steatosis has also been assessed.

The exact amount of hepatic fat content can be determined, without subjecting the humans to radiation, by using liver spectroscopy. We have used this technique in earlier studies with good results. We now want to study humans in a randomized manner during two months in which the participants between regular meals either consume fruits or matched to the same extra caloric intake from a source with less fructose content, nuts. The aim of the trial is to study metabolic effects of the supplements on risk markers for diabetes and cardiovascular diseases with blood sampling, liver spectroscopy and measurement of basal metabolic rate by indirect technique. We also want to assess effects on teeth and on quality of life by using questionnaires.

**Study design**

Recruitment of 28 men and women who are randomized to 7 kCal/kg body weight per day of nuts or fruit added on top of regular food intake.

The fruit or nuts are bought and chosen by the participants and the costs are reimbursed consecutively based on receipts. The participants keep notes of daily intake. We will ask them to preferably consume traditional fruit and nuts such as bananas, apples and pears (the study will run in the autumn) and to choose eco-friendly fruits.

The nuts are also chosen by the participants and will be reimbursed consecutively as are the fruits. We will recommend hazelnuts and almonds and also peanuts which will be regarded as nuts.

Before study start we will obtain medical history and physical examination. Routine blood tests will be obtained (hemoglobin, transaminases, creatinine, thyroid hormones etc). Serious deviations in these analyses will constitute exclusion criteria. Students that are supervised by the study organizers can not be recruited. The blood samples are drawn at study baseline and at the end of the study. Indirect calorimetry, to determine metabolic rate, is also done at these two time points as are determinations of hepatic fat content. The liver spectroscopy analysis is analyzed blindly by the technician. Teeth status is determined by a dentist at baseline and at the end of the study to judge gingivitis and bacterial afflictions (no x-ray). A small device that registers movements will be carried on a belt for three days to investigate if physical activity is affected by the interventions (accelerometry).

**The investigations at start and at the end of the trial with randomization to nuts or fruit as snacks between meals are:**

Fasting blood samples

Hemoglobin, liver tests, electrolytes, inflammation, vitamin-C, blood lipids, insulin, glucose, uric acid, thyroid hormones.

Liver spectroscopy

Indirect calorimetry

3-day diet registration

Teeth status (gums)

QoL by questionnaires

Accelerometry for three days

Anthropometric data (height, weight, abdominal circumference, blood pressure etc)

The participants will be informed about all tests and will receive 2000 SEK for the participation, after tax reduction.

In an earlier investigation of increased caloric intake from soda (Coke, half glucose, half fructose from the sugars in the drink) showed more than double increase (+120%) of hepatic steatosis based on the + 500 kCal/day for 6 months [1]. We have 80% power to detect a 50% increase of liver steatosis by MR spectroscopy provided that the participants do not have hepatic steatosis at baseline. The theory is that the nut-group will have reduced hepatic fat levels [1], so in that regard the power is stronger to detect differences between the groups.

Fredrik H Nystrom

Professor IMH, consultant in endocrinology

**Reference**

1. Maersk M, Belza A, Stodkilde-Jorgensen H, Ringgaard S, Chabanova E, Thomsen H, et al. Sucrose-sweetened beverages increase fat storage in the liver, muscle, and visceral fat depot: a 6-mo randomized intervention study. The American journal of clinical nutrition. 2012;95(2):283-9. Epub 2011/12/30. doi: ajcn.111.022533 [pii]10.3945/ajcn.111.022533. PubMed PMID: 22205311.
