# Supplementary material for: A Randomized Study of the Effects of Additional Fruit and Nuts Consumption on Hepatic Fat Content, Cardiovascular Risk Factors and Basal Metabolic Rate
Source: PLoS One. 2016 Jan 20;11(1):e0147149. doi: 10.1371/journal.pone.0147149 (PMC4720287; doi:10.1371/journal.pone.0147149)
Supplement: S3 File — (DOC) [file pone.0147149.s003.doc]

**Forskningsprogram: Effekter av frukt eller nötter som mellanmål på riskfaktorer för hjärt- kärlsjukdom**

Frågeställning:

**Leder ett intag av frukt som mellanmål till mindre fördelaktiga metabola förändringar än ett intag av nötter?**

**Bakgrund**

Många personer äter mellanmål i syfte att döva hungern mellan huvudmåltiderna. Detta är också ett råd som ges av många dietister enligt riktlinjer från t.ex. Livsmedelsverket. Ofta föreslås att man äter frukt som mellanmål för att få i sig t.ex. vattenlösliga vitaminer, det finns nu flera företag som säljer just fruktkorgar vilka sponsras av arbetsgivare så att arbetstagaren kan få en frukt om dagen. Nyare data talar för att man skall begränsa sockerintaget för att minska fetmaepidemin, men denna debatt rör huvudsakligen föda som innehåller tillsatt socker. Fruktdrycker anses av vissa fortsatt vara ”nyttigt” medan andra ifrågasätter all former av söt dryck och menar att dessa kan vara skadliga och öka risken för fetma. Flera moderna studier talar för att just fruktsocker, fruktos, som förekommer i frukt, innebär en ökad risk för metabola bieffekter inklusive minskad känslighet för insulin (insulinresistens) och framkallande av leverförfettning. Än så länge har intresset att studera det mer specifika effekterna av söta frukter, som man intar utan att först göra juice, varit mycket begränsat. Man kan tänka sig att fiberinnehållet i frukten som äts, tuggas, som hel frukt utgör ett skydd mot metabola bieffekter av sådant intag, men det finns inte specifikt studerat enligt vad vi kan finna i vetenskaplig litteratur, i någon randomiserad studie där också leverförfettning undersöks.

Med så kallad leverspektroskopi kan mängden fett i levern fastställas utifrån strålningsfri magnetresonanstomografi (MR) teknik. Vi har använt denna teknik i tidigare undersökningar med gott resultat i studier på människa. Vi önskar nu göra en randomiserad studie under 2 månader där deltagare lottas till mellanmål i form av frukt eller till motsvarande kaloriintag från en källa med mindre fruktos, nötter. Studien syftar fr.a. till att undersöka metabola effekter av dessa två kosttillskott på riskfaktorer för diabetes och hjärt- kärlsjukdom med blodprover, mätning av ämnesomsättning samt leverspektroskopi för bestämning av mängden fett i levern. Enkäter kommer också användas för att se om vi får effekter på livskvalitet och vi planerar också undersöka om tandstatus påverkas av de olika mellanmålen.

**Studieupplägg**

Rekrytering av ca 28 män och kvinnor som lottas till 7 kCal/kg kroppsvikt intag av nötter eller frukt som mellanmål.

Frukten får väljas och inköpas av deltagarna själva för att öka följsamhet. Kostnadsersättning mot kvitto i efterhand. Dagbok över fruktintag förs av deltagarna. Vi kommer i första hand önska att man konsumerar vanligt förekommande frukt såsom äpplen och päron (studien görs på hösten), gärna ekologist odlat.

Nötsort får väljas och inköpas av deltagarna själva för att öka följsamhet. Kostnadsersättning mot kvitto i efterhand. Dagbok över nötintag förs av deltagarna. Vi kommer i första hand önska att man konsumerar vanligt förekommande nötter såsom valnötter, hasselnötter etc. Jordnötter räknas i detta fall också som en nöt (trots att det är en baljväxt egentligen).

Innan studien startas tas anamnes och status, och allmän rutinprovtagning (blodvärde, levervärden/prover, njurar, ämnesomsättning etc) för att se så att ingen allvarlig sjukdom föreligger som kan påverka studieanalyserna. Studenter i beroendeställning kan inte rekryteras som deltagare. Prover på ämnesomsättning samt rutinprover upprepas efter 8 veckor (avslut). Basmetabolism med registrering av koldioxidproduktion samt syrgasupptag utföres därtill i vila vid dessa tidpunkter samt magnetresonanstomografi (MR) för bestämning av leverfettmängd samt dessutom kroppssammansättning (muskler och fettfördelning). Den separata individuella MR analysen sker blindat så långt detta är tekniskt möjligt. Tandstatus utföres av tandläkare före och efter perioden, i syfte att fr.a. klassa graden av eventuell tandstens och tandköttsinflammation (ingen röntgen). Tandläkaren informeras inte om vilken grupp deltagaren lottats till. Tandköttsinflammation uppskattas med tandfickdjupet på varje tand. En liten dosa bärs i bältet under tre dagar för att ge information om graden av fysisk aktivitet och om detta påverkas av interventionen (accelerometer).

**Undersökningar och prover före och efter (i slutet av perioden) lottning till nötter eller frukt som mellanmål:**

Blodprover i fastande

Levervärden, blodvärde, elstatus, inflammationsprover, halt av c-vitamin i blodet, blodfetter, insulin, glukos, urat, sköldkörtelprover.

MR, leverspektroskopi

Indirekt kalorimetri

Kostregistrering under 3 dagar

Tandstatus (tandfickors djup)

Enkäter (SF 36)

Mätning av fysisk aktivitet (accelerometer under 3 dagar)

Antropometri (längd, vikt, midjeomfång, blodtryck mm).

Deltagarna får ta del av prover och undersökningar efter studien och får också 2000 kr i ersättning (efter skatteavdrag) för att vara med.

En tidigare undersökning av extra kaloriintag från läsk (Cocacola, hälften glukos hälften fruktos i sockret) visade en dryg fördubbling (+120%) av leverförfettning baserat på ca 500 Kcal extra/dag i 6 månader . Vi har en power på 80% att detektera en stegring på 50% i leverförfettning via MR spektroskopi hos personer som inte har uttalad steatos från början. Men teorin är att nötgruppen får minskad steatos (det såg man en klar trend till att få i en studie av mjölk med relativt mycket fett i ) så därmed är power mellan grupperna för studien en skillnad i interventionerna starkare.

Fredrik Nyström

professor IMH, överläkare i endokrinologi

**Referenser**

[1] Maersk, M, Belza, A, Stodkilde-Jorgensen, H, Ringgaard, S, Chabanova, E, Thomsen, H, Pedersen, S B, Astrup, A, and Richelsen, B. Sucrose-sweetened beverages increase fat storage in the liver, muscle, and visceral fat depot: a 6-mo randomized intervention study. Am J Clin Nutr 2012;95:283-9.
