# Supplementary material for: A Randomized Study of the Effects of Additional Fruit and Nuts Consumption on Hepatic Fat Content, Cardiovascular Risk Factors and Basal Metabolic Rate
Source: PLoS One. 2016 Jan 20;11(1):e0147149. doi: 10.1371/journal.pone.0147149 (PMC4720287; doi:10.1371/journal.pone.0147149)
Supplement: S4 File — (DOCX) [file pone.0147149.s004.docx]

**APPLICATION FOR ETHICAL VETTING OF RESEARCH INVOLVING HUMANS** 1

**APPLICATION FOR ETHICAL VETTING OF RESEARCH INVOLVING HUMANS**

**For information concerning the application, see *Guide to the application (www.epn.se)***

IT IS NOT PERMITTED TO USE THE ENGLISH APPLICATION FORM OR TO FILL IN THE APPLICATION IN ENGLISH. THE SWEDISH APPLICATION FORM MUST BE USED.

**Depending upon what research the application concerns, the information asked for below will have various degrees of relevance. In cases of changes to a previously approved application, see *Guide to the application*.**

**To the Regional Ethical Review Board in:** *Linköping, Sweden*

State the Regional Ethical Review Board to which the entity principally responsible for the research belongs. For a list of boards see *www.epn.se*.

Date fee paid: April 4 2014

Please note that an application is never complete (and thus able to be processed) until the form is correctly filled in and the fee has been paid.

**Project title: Does extra intake of fruit lead to less advantageous metabolic effects than nuts?**

Give a descriptive title in Swedish for laymen, without using confidential information. Where suitable, for instance in cases of clinical trials of medicinal products, also state the identity of the project and the number, date, version etc. of the project/research plan (protocol or testing plan). In the case of changes to a previously approved application, see Guide to the application.

Project number/identity: Version number:

EudraCT nr (for clinical trials of medicinal products):

***Information to be completed by the Regional Ethical Review Board***

Application complete: Case number:

Request for additional information concerning the application: Requested information received:

Date of decision: Date processed:

**The application concerns (also applies when an advisory statement is requested): APPLICATION FOR ETHICAL VETTING OF RESEARCH INVOLVING HUMANS**

research in which only one responsible research body participates (5 000 kr) **yes**

research in which more than one responsible research body participates (16 000 kr)

research in which more than one responsible research body participates,

but in which all the researchers or subjects of research have an immediate

link to only one of the responsible research bodies (5 000 kr)

only processing personal data (5 000 kr)

(When only existing population registers, e.g. national databases, will be used)

research involving clinical trials of medicinal products (16 000 kr)

changes to a previously approved application in accordance with p. 4 of Statute

(2003:615) concerning the Ethical Review of Research Involving Humans (2 000 kr)

If the board decides that the legislation concerning ethical review is not applicable to the study/research project, is an advisory statement wanted? (See p. 4a and 4b in Statute 2003:615 and Guide to the application)

Yes: No:

**1. Information concerning the body principally responsible for the research etc.**

**1:1 The responsible research body** (See p. 1:1 in Guide to the application)

The application for ethical vetting of research is to be made by the responsible research body. *By responsible research body is meant a government authority or a physical or legal entity under whose auspices the research is conducted.*

Name: *Department of Endocrinology Linköping University Hospital.*

Address*: 581 85 Linköping*

**1:2 Qualified representative of the body principally responsible for the research**

A qualified representative can be, for example, head of department, head of unit or head of operations. The responsible research body is to decide itself by internal consultation, delegation, or by means of power of attorney, who is qualified to act as a representative of the body.

Name: Official title: *Anders Fernström*

Address: *Department of Endocrinology Linköping University Hospital, 581 85 Linköping*

**1:3 The researcher/s primarily responsible for the conduct of the project**

**(principal contact person/s)** (See p. 9, annex 10 and p. 1:3 in Guide to the application)

Note! It is the principal researcher who is responsible for ensuring that others carrying out the project have sufficient competence (scientific as well as clinical) and, in the case of clinical trials of medicinal products, are **APPLICATION FOR ETHICAL VETTING OF RESEARCH INVOLVING HUMANS** familiar with “Good Clinical Practice” (GCP). When work is being carried out by doctoral candidates, the principal supervisor is the principal researcher who bears the main responsibility for the conduct of a project, as a rule.

Name: *Fredrik H Nyström Official title: Professor, consultant*

Address: *Department of Endocrinology Linköping University Hospital 581 85 Linköping*

E-mail: *fredrik.nystrom@lio.se*

Telephone: *0101037749*

Mobile phone: *0736 569303*

**1:4 Other participants** (See p. 9, annex 1 and p. 1:4 in Guide to the application)

Other responsible research bodies, plus researchers responsible for completing the project locally (principal contacts) are to be listed here or in an annex stating names and addresses (see p. 9, annex 1).

*Hans Guldbrand, Torbjörn Lindström.*

**1:5 Describe access to necessary resources during the course of the project**

(See p. 9, annex 9 and p. 1:5 in Guide to the application)

List the person/s (head of department, director of department etc.) who will be responsible for the research participants’ safety at all units/clinics where they will participate. Testimonials from this/these responsible party/ies must be appended (see p. 9, annex 9). The testimonial/s should state that the required economic, structural and personnel resources are available to guarantee the safety of the research participants.

*Fredrik Nystrom has personnel at the University Hospital and at Linköping University. Nystrom has responsibility for safety of participants.*

**1:6 Applications/notifications to other authorities as applicable**

(See p. 1:6 in Guide to the application) **Submitted Date**

a) Clinical testing of medicinal products: Swedish Medical Products Agency _________ *NA*

b) Handling of personal data concerning genetic dispositions: Swedish Data Inspection Board _________*NA*

c) Establishment of a biobank: Swedish National Board of Health and Welfare _________ *NA*

d) Research involving ionising radiation: :radiation protection committee(s) _________ *NA*

**2. Information concerning the project**

**2:1 Summary of the research project** (See p. 9, annex 2 and p. 2:1 in Guide to the application) **APPLICATION FOR ETHICAL VETTING OF RESEARCH INVOLVING HUMANS** 4

The description must be comprehensible to all board members. It is therefore advisable to avoid specialist terminology. State the background and the purpose of the study, together with the scientific question(s) for which answers are being sought. State the most important variables for the investigation. State what advances in knowledge can be expected as a result of the project and what the significance of these might be. State if it is a study of records, if the research is commissioned, etc. Detailed information in the research plan, protocol or programme that is intended for specialists must be appended as an annex (see p. 9, annex 2). For information on what to include in the research plan/protocol, p. 2:1 in Guidance to the application. Include when the data collection is expected to be complete. A more detailed description that is *intended for laymen* concerning the implementation of the study can, if needed, be appended to the obligatory research plan intended for experts.

*Many people eat snacks in between regular meals to reduce hunger. It is often suggested that fruit is a good alternative for such snacking due to high content of vitamins. There are many companies that supply work places with fruit baskets that are paid by employers so that employees can eat one fruit a day. New data suggest that intake of sugars should be limited to reduce the obesity epidemic, but this debate mainly concerns added sugars in food. Many modern studies suggest that fructose, often found in fruit, is linked with increased risk for metabolic side effects including reduced insulin sensitivity to glucose uptake in the insulin-responsive cells, i.e. so called insulin resistance. According to some controversial studies, fructose can induce hepatic steatosis in human. So far, the interest to study effects of whole fruits, rather than fruit juice, has been very limited, in these respects. It could be argued that the fibre content in fruit could constitute a protection against the risks with a high intake of the sugars in fruit, but according to our knowledge this has not been tested in humans in a randomized manner.*

*With liver spectroscopy the exact amount of hepatic fat content can be established without subjecting the humans to radiation. We have used this technique in earlier studies with good results. We know want to study humans in a randomized manner during two months in which the participants either consume nuts or fruits as snacks to be consumed in between meals. The aim of the trial is to study effects of the supplements on cardiovascular risk markers including blood samples, liver spectroscopy and on basal metabolic rate by indirect technique. We also want to assess effects on teeth and on quality of life by using questionnaires.*

*Study design*

*Recruitment of 28 men and women who are randomized to 7 kCal/kg body weight per day of nuts or fruit added on top of regular food intake.*

*The fruit or nuts are bought and chosen by the participants and the costs are reimbursed consecutively based on receipts. The participants keep notes of daily intake. We will ask them to preferably consume traditional fruit and nuts such as bananas, apples and pears (the study will run in the autumn) and to choose eco-friendly fruits.*

*The nuts are also chosen by the participants and it will be reimbursed consecutively as are the fruits. We will recommend hazelnuts and almonds and also peanuts which will be regarded as nuts.*

*Before study start we will obtain medical history and physical examination. Routine blood tests will be obtained (hemoglobin, transaminases, creatinine, thyroid hormones etc). Serious deviations in these samples will constitute exclusion criteria. Students that are supervised by the study organizers can not be recruited. The blood samples are drawn at study baseline and at the end of the study. Indirect calorimetry, to determine metabolic rate, is also done at these two time points as are determinations of hepatic fat content. The liver spectroscopy analysis is performed blindly by the technician. Teeth status is determined by a dentist at baseline and at the end of the study to judge gingivitis and bacterial affections (no x-ray).*

*The investigations at start and at the end of the trial are:*

*Blood tests (liver, hemoglobin, electrolytes, inflammation, vitamin-C, blood lipids, insulin, glucose, uric acid).*

*Liver spectroscopy*

*Indirect calorimetry*

*3-day diet registration*

*Teeth status (gums)*

*QoL by questionnaires*

*Accelerometry for three days*

*Anthropometric data (height, weight, abdominal circumference, blood pressure etc)*

*The participants will get information about all tests and will receive 2000 SEK for the participation, after tax reduction.*

**2:2 What is/are the primary scientific question(s) forming the basis of the design of the**

**project?**

If the project can be described as testing a hypothesis, state the primary hypothesis and the secondary hypothesis if there is one. Referral can be made to more detailed information for experts in the form of an appended research plan in accordance with 2:1.

*Does extra intake of fruit as snack lead to less advantageous effects on CVD risk factors and diabetes than the same amount of extra energy from nuts (with high content of fat and protein rather than sugars and vitamins as in fruit)?*

**2:3 State the results from relevant animal experiments (clinical trials)**

If animal experiments have not been carried out, the reasons for this must be given.

*Not applicable. Animals have different ways of life and a different metabolism than humans.*

**2:4 Give an overview of the examination procedures used, data collection and the nature of**

**the data** (See p. 9, annex 5 and p. 2:4 in Guide to the application)

It should be clear from the description how it is planned to complete the project. Describe the nature of data collected. Describe how the reliability of the data is ensured (e.g. quality control/monitoring). When questionnaires and interviews are used, the procedure used should be described and, for example, the content of questions and how conclusions are drawn. Questionnaires and rating scales must be appended (see p. 9 annex 5). For medical research it should be stated, for example, the types of intervention, the methods of measurement, the number of visits, the time required each time research is carried out and the means used to administer any medicinal products and/or isotopes and the amount of blood samples taken (including the accumulated amount when multiple testing takes place). State also if the examination procedure etc. differs from routine clinical measures, and if so, in what way. If a treatment is being studied for the first time on humans, this should be stated and the relevant safety measures described. State which procedure can be required to administer the prospective treatment after the conclusion of the study. State the procedures used to collect biological material. Account for data sources and procedures when processing personal data. For more detailed information, referral can be made to the appended research plan. **APPLICATION FOR ETHICAL VETTING OF RESEARCH INVOLVING HUMANS** 5

*The investigations at start and at the end of the trial are:*

*Blood tests (liver, hemoglobin, electrolytes, inflammation, vitamin-C, blood lipids, insulin, glucose, uric acid).*

*Liver spectroscopy*

*Indirect calorimetry*

*3-day diet registration*

*Teeth status (gums)*

*QoL by questionnaires*

*Accelerometry for three days*

*Anthropometric data (height, weight, abdominal circumference, blood pressure etc)*

**2:5 Describe how collected biological material is to be stored in a biobank**

(See p. 2:5 in Guide to the application)

*By biobank is meant biological material that has been collected and retained from one or more persons, the origins of which can be traced to the person or persons from which it originates and is being kept pending further notice or for a certain period of time.* Describe where and how each sample to be retained will be kept, the coding procedures and what conditions apply when material is released. State who the person responsible for the biobank is.

*Blood for later analysis of lipids and hormones stored in -80C freezer situated at pharmacological department, U.S for a couple of months. Fredrik Nystrom is responsible for the blood samples for analysis. Data will be processed anonymously. Responsible for biological material is the same as listed above.*

**2:6 Report the necessary resources during project implementation**

Specify who is responsible (director or equivalent) for research subjects' safety at all units / clinics where research subjects will participate. Certificate from those responsible must be attached (see p. 9 Appendix 9). The certificate must state that the requisite economic, structural and human resources are available to ensure the safety of research subjects.

*List with codes of participants to use for deciphering coded data in original files is kept by F Nystrom. No medical procedures are performed so no medical journal is kept. Original data are kept at the University hospital, unidentifiable data are kept at the University. Laboratory samples indicative of disease are handled clinically according to routine at the clinic of Endocrinology outside of the trial with F Nystrom as the responsible physician.*

**2:7 Documentation, data protection and record-keeping** (See p. 2:7 in Guide to the application)

State how the examination procedures and any operations that take place are to be recorded. State if audio and video recordings are to be used. If the material is to be coded, state the procedure: who will retain the code list /code-key in safe-keeping and which person or persons have access to them; where they are being kept and for how long; and if the material will be rendered anonymous or destroyed. Describe how accessible the data material is, how it is kept and how the requisite confidentiality is attained.

*Code list of linking anonymous data with actual data / personal number performed by PI Fredrik Nyström. No medical work and no medical procedures are performed, and thus there is no medical record keeping in the study. The data will be saved at the University Hospital. Depersonalized data files will be handled within the University (HU). If there are samples that arises suspicion of disease this will be handled in the usual clinical manner at the clinic with usual record keeping and investigation outside the study where Fredrik Nystrom has the responsibility.*

**2:8 Describe previous experience (your own and/or others’) of the procedure, technique or**

**treatment used**

It is particularly important that the risks of complications are clearly accounted for and that the relevant publications are listed. When patients are to be given a new form of treatment, such as a pharmaceutical product, it should be stated how many patients (with the complaint in question or another one), had previously been given the proposed treatment, dosage of a pharmaceutical product (or other dosage) and over which treatment periods the treatment has been studied.

*We have conducted studies in healthy volunteers where similar investigations were carried out. Participants are not exposed to risks other than giving blood samples.*

**3. Information about the research participants**

**3:1 How are research participants chosen?** (See p. 9, annex 3 and p. 3:1 in Guide to the application)

*By research participant is meant a living person who is the subject of the research.* State the selection criteria (inclusion and exclusion). Give an account of the manner in which the researcher will get in contact with/become aware of suitable participants for the research. If advertising is to take place, the advertising material must be submitted as an annex (see p. 9 annex 3). If, for example, children or people who, temporarily or permanently, are not capable of themselves giving their informed consent are to participate in the project, this is to be specifically justified. If certain groups are to be excluded from the project, this is to be specifically justified. **APPLICATION FOR ETHICAL VETTING OF RESEARCH INVOLVING HUMANS** 6

*Inclusion criteria: Healthy participants with age above 18 years.*

*Exclusion criteria: Significant liver disease. Physical or mental problems that make participation difficult to implement or to understand instructions.*

**3:2 State the relationship between the researchers/leader of the research and those participating in the research**

Person administering the treatment (e.g. doctor, psychologist, physiotherapist) - person participating in the research (e.g. patient, client)

***Researcher- healthy participant***

**3:3 State the statistical foundation with respect to the size of the population(s) and/or**

**material(s) studied** (See p. 3:3 in Guide to the application)

Give an account of the statistical power, the so-called power-calculation, or give an account of equivalent considerations which clarify the study's ability to answer the questions posed.

*Based on our earlier studies of diet we find 28 participants to give clinically meaningful statistical power, we can detect a 50% increase in hepatic fat by fruit with 80% power.*

**3:4 State if participants in the research may be included in several studies, either**

**simultaneously or in another study or other studies closely linked to this one. If so, what**

**kind of research?** (See p. 3:4 in Guide to the application)

*No it is not intended.*

**3:5 What insurance cover is there for research participants taking part in the project?**

It is the responsibility of the entity principally responsible for the research to check that existing insurance policies cover any injuries that may arise in connection with the research.

*We can not see that the study means any real risks, but hospital insurance covers complications of venous sampling.*

**3:6 What financial remuneration or other benefits are participants in the research entitled**

**to and when is this to be paid? (A more detailed description can be submitted as an annex.)** (See p. 9, annex 11 and p. 3:6 in Guide to the application)

Compensation for discomfort and inconvenience - amount (before tax):

Compensation for income from employment No

Allowance for travelling expenses No

Exemption from costs of pharmaceutical products No

Exemption from other costs - which? No

Other benefits - which? Compensation for sampling: *2000 SEK in total.*

When is compensation paid? *After completion of the study.*

No compensation to be paid **APPLICATION FOR ETHICAL VETTING OF RESEARCH INVOLVING HUMANS** 7

**4. Information and consent**

**4:1 The procedure involved and the content of the *information* that is given when subjects**

**are asked to participate in the research** (See p. 9, annex 4 and Information for research participants)

In accordance with section 16 of the Act (2003:460) concerning the Ethical Review of Research Involving Humans, research participants are to be informed of the overall plan for and purpose of the research, the methods that will be used, the consequences and risks the research can entail, who the entity principally responsible for the research is, that participation is voluntary, and that they can end their participation at any time. Describe how and when the information is given and what it contains. Indicate who provides the information. Brief and easily comprehensible written information should normally be given. This written information must be attached to the application (see p. 9 annex 4). If no information or incomplete information is given, detailed reasons for this must be given.

*The subjects will be informed orally and in writing of the study by representative who meets them for recruitment purposes.*

**4:2 How is *consent* to be obtained and from whom?** (See Information for research participants)

Describe the procedure: who asks, when this takes place and how the consent is documented. Exhaustive

documentation is particularly important, not only when children or people with a diminished ability to make

decisions participate in the study but also when the study involves a group or groups such as school classes, associations, organisations, companies, church communions, congregations, or groups that interact within social media.

*Documentation by written informed consent.*

**5. Considerations in the light of research ethics**

**5:1 Describe the risks that participation might entail**

This could mean, for example, physical injury, pain, discomfort or other violations of personal integrity that the

project entails or can entail, both long-term and short-term. State not only which measures been taken to prevent the risks mentioned above, but also what preparations have been made to deal with these complications. Give an account of the methods that will be used to investigate, register and report undesirable events.

*Participants are exposed to minimal risks. As we only will recruit subjects who are aware of the risks of bruises of blood sampling we think eating snacks for 8 weeks in the form of nuts or fruit should be free of risks.*

**5:2 Describe the predictable benefit for the people participating in the research who are**

**part of the project (applies especially to treatment)**

*Experience in participating in a research project, knowledge of the levels of various cardiovascular risk factors / blood tests/ MRI of liver and how they are affected by different kinds of diets.*

**5:3 In a broader perspective, identify and specify which ethical problems, such as risk**

**versus benefit, can arise as a part of or as a result of the project** (See p. 5:3 in Guide to the application)

*We believe that the scientific value of the study and interest in the individual participant is greater than the very small risk exposure in patients.*

**APPLICATION FOR ETHICAL VETTING OF RESEARCH INVOLVING HUMANS** 8

Here, for example, an account can be given of which groups (besides the research participants within the research project) can be identified or helped as a result of the study.

*In the longer term differences between the effects of the snacks tested could affects advice given to stay healthy. But this depends on our final findings.*

**6. Presenting the results**

**6:1 How are both the entity principally responsible for the research and research**

**collaborators guaranteed access to data (to be stated when the research is commissioned) and who is responsible for processing data and writing reports?** (See p. 6:1 in Guide to the application)

*Fredrik Nystrom is responsible for data collection and for reporting in scientific papers in peer-reviewed international journal.*

**6:2 How will the results be made publicly available? Will the study be sent for publishing in**

**a journal or published in some other manner?** (See p. 6:2 in Guide to the application**)**

State the format in which it is planned to make the results public and indicate the time frame that is applicable.

*Scientific reports in peer-reviewed international journals.*

**6:3 In what manner will the right to integrity of those participating in the research be**

**guaranteed when the material is made public or is published?**

Will results be shown at a statistical group level? Describe the procedures or the methods used for concealing identities or ensuring anonymity.

*Results are presented for groups. All presentations are completely anonymous.*

**7. Reporting the financial circumstances and dependencies**

**The purpose of the accounts to be given in points 7:1-7:3 is to clarify all direct or indirect circumstances that could possibly affect the researcher's relationship to the persons participating in the research (during the process of information, consent or implementation, for example).**

**7:1 When the research is commissioned**

State who the commissioning body is: for example a company (when there is clinical testing of pharmaceuticals or the testing of other new products, for example) an organisation or an authority.

Name: Contact for the commissioning body: Not applicable.

Address: Telephone/mobile phone: **APPLICATION FOR ETHICAL VETTING OF RESEARCH INVOLVING HUMANS** 9

State the relationship between the responsible research body for the research/participating researchers and the commissioning body the research (employee/employer, for example).

**7:2 Give an account of any financial agreements with a responsible body or any other financiers**

**(name, amount)** Not applicable.

Where the clinical testing of pharmaceuticals is concerned, reference should be made to agreements entered into with the responsible health-care body. Similar agreements may occur when other research is commissioned and should be accounted for in the same way. Separate agreements with the person or persons who are to carry out the research are also to be presented. State the amount that is allocated for the study/compensation to the clinic/person carrying out the research and what the compensation is to cover. Any sums given to persons participating in the research should also be accounted for here.

*Not applicable.*

**7:3 Give an account of the interests of the responsible research body, the principal researcher and of participating researchers**

An account is to be given here of, for example, ownership of shares, employment status, consultancy work for companies providing finance and any companies owned by the researchers that could benefit financially, directly or indirectly, as a result of the research.

*There are no known such vested interests in this study.*

**8. Signatures**

Authorized representative for the responsible research body which is making the application, according to 1.2

Place: Date:

Signature: __________________________________________________________________

Clarification of signature:

Official title:

The undersigned researcher who is carrying out the project (principal researcher) according to.1:3 hereby certifies that the research will be carried out in accordance with the application.

Place: Date:

Signature: _________________________________________________________________ **APPLICATION FOR ETHICAL VETTING OF RESEARCH INVOLVING HUMANS** 10

Clarification of signature:

Official title: **APPLICATION FOR ETHICAL VETTING OF RESEARCH INVOLVING HUMANS** 11
