# Supplementary material for: A Randomized Study of the Effects of Additional Fruit and Nuts Consumption on Hepatic Fat Content, Cardiovascular Risk Factors and Basal Metabolic Rate
Source: PLoS One. 2016 Jan 20;11(1):e0147149. doi: 10.1371/journal.pone.0147149 (PMC4720287; doi:10.1371/journal.pone.0147149)
Supplement: S5 File — (DOC) [file pone.0147149.s005.doc]

*Beslutad 2013-03-04*

**ANSÖKAN OM ETIKPRÖVNING AV FORSKNING SOM AVSER MÄNNISKOR**

# Information till ansökan, *se Vägledning till ansökan (*[*www.epn.se*](http://www.epn.se/)*)*

**Beroende på vilken forskning som ansökan gäller kommer de uppgifter som efterfrågas att ha olika relevans. Vid ändring av tidigare godkänd ansökan, se *Vägledning till ansökan*.**

**Till Regionala etikprövningsnämnden i:** Linköping

Den regionala etikprövningsnämnd till vars upptagningsområde forskningshuvudmannen hör, se respektive nämnd *(*[*www.epn.se*](http://www.epn.se/)*)*.

Avgift inbetald datum: 8/4 2014

Observera att en ansökan aldrig är komplett och därmed kan behandlas förrän blanketten är korrekt ifylld och avgiften är betald.

**Projekttitel:** Leder ett intag av frukt som mellanmål till mindre fördelaktiga metabola förändringar än ett intag av nötter?

Ange en beskrivande titel på svenska för lekmän. Titeln ska ej innehålla sekretesskyddad information. Ange också i förekommande fall, t.ex. vid klinisk läkemedelsprövning, projektets identitet, forskningsplanens/protokollets nummer, version, datum. Vid ändring av tidigare godkänd ansökan, se [Vägledning till ansökan](http://www.epn.se/media/45470/v_gledning_till_ans_kan_2012-03-27.doc).

Projektnummer/identitet:       Version nummer:       EudraCT nr (vid läkemedelsprövning):

## **Uppgifter som fylls i av den regionala etikprövningsnämnden**

Ansökan komplett: Dnr:

Begäran om ytterligare information (i sak): Begärd information inkommen:

Beslutsdatum: Expeditionsdatum:

**Ansökan avser (gäller även vid b****egäran om rådgivande yttrande):**

Forskning där endast en forskningshuvudman deltar (5 000 kr)

Forskning där mer än en huvudman deltar (16 000 kr)

Forskning där mer än en forskningshuvudman deltar, men där samtliga

forskningspersoner eller forskningsobjekt har ett omedelbart

samband med endast en av forskningshuvudmännen (5 000 kr)

Endast behandling av personuppgifter (5 000 kr)

(När enbart redan befintliga personregister ska användas, t. ex. nationella databaser)

Forskning som gäller klinisk läkemedelsprövning (16 000 kr)

Ändring av tidigare godkänd ansökan enligt 4 § förordning (2003:615) om

etikprövning av forskning som avser människor (2 000 kr)

Om nämnden finner att forskningsprojektet inte faller inom etikprövninglagens tillämpningsområde

önskas ett rådgivande yttrande. (Se [4a och 4b §§ i förordning 2003:615](http://www.epn.se/media/8604/2003_615_4ab.doc) och [Vägledning till ansökan](http://www.epn.se/media/45470/v_gledning_till_ans_kan_2012-03-27.doc))

Ja:  Nej:

**1. Information om forskningshuvudman m.m.**

**1:1 Forskningshuvudman** ([Se p. 1:1 i Vägledning till ansökan](http://www.epn.se/media/43343/vta_p1_1.doc))

Ansökan om etikprövning av forskning ska göras av forskningshuvudmannen. *Med forskningshuvudman avses en statlig myndighet eller en fysisk eller juridisk person i vars verksamhet forskningen utförs.*

Namn: Endokrinmedicin, Universitetssjukhuset i Linköping

Adress: HU, 581 85 Linköping

**1:2 Behörig företrädare för forskningshuvudmannen**

Behörig företrädare är t.ex. prefekt, enhetschef, verksamhetschef. Forskningshuvudmännen bestämmer själva, genom interna arbets- och delegationsordningar eller genom fullmakt, vem som är behörig att företräda forskningshuvudmannen.

Namn: Anders Fernström Tjänstetitel: Klinikchef, ÖL, docent

Adress: Universitetssjukhuset i Linköping, 581 85 Linköping

**1:3 Forskare som är huvudansvarig för genomförandet av projektet (kontaktperson)**(Se [p. 9 bil. nr 10 och p. 1:3 i Vägledning till ansökan](http://www.epn.se/media/43346/v_gledning_till_ans_kan_p1_3.doc))

Observera! Den som är huvudansvarig forskare ansvarar för att andra medverkande som ska genomföra projektet har tillräcklig kompetens (vetenskaplig och klinisk) och vid läkemedelsprövning har tillräcklig kunskap om ”Good Clinical Practice” (GCP). Vid doktorandstudier är som regel handledaren huvudansvarig forskare.

Namn: Universitetssjukhuset i Linköping, 581 85 Linköping Tjänstetitel: Professor, ÖL

Postadress: HU, 581 85 Linköping

E-postadress: fredrik.nystrom@lio.se

Telefon: 0101037749

Mobiltelefon: 0736569303

**1:4 Andra medverkande** ([Se p. 9 bil. nr 1 och p. 1:4 i Vägledning till ansökan](http://www.epn.se/media/43349/v_gledning_till_ans_kan_p1_4.doc))

Övriga deltagande forskningshuvudmän samt forskare ansvariga för att lokalt genomföra projektet (kontaktpersoner) anges här eller i bilaga med namn och adresser (se p. 9 bil. nr 1).

Hans Guldbrand, Torbjörn Lindström

**1:5 Redovisa tillgång till nödvändiga resurser under projektets genomförande**

(Se p. 9 bil. nr 9 och [p. 1:5 i Vägledning till ansökan](http://www.epn.se/media/43352/v_gledning_till_ans_kan_p1_5.doc))

Ange vem/vilka som har ansvaret (prefekt, verksamhetschef eller motsvarande) för forskningspersonernas säkerhet vid alla enheter/kliniker där forskningspersoner ska delta. Intyg från dessa ansvariga *ska* bifogas (se p. 9 bil. nr 9). Av intyget ska framgå att erforderliga ekonomiska, strukturella och personella resurser finns tillgängliga för att garantera forskningspersonernas säkerhet.

Personella resurser på Universitetet och i Landstinget finns för projektet, se intyg. Fredrik Nyström har ansvaret för försökspersonernas säkerhet

**1:6 Ansökan/anmälan till andra myndigheter i vissa fall**

([se p. 1:6 i Vägledning till ansökan](http://www.epn.se/media/43355/v_gledning_till_ans_kan_p1_6.doc))  **Insänd Datum**

a)Vid klinisk läkemedelsprövning: Läkemedelsverket

b)Vid inrättande av biobank: Socialstyrelsen

c)Vid undersökning omfattande joniserande strålning: Strålskyddskommitté

### 2. Uppgifter om projektet

**2:1 Sammanfattande beskrivning av forskningsprojektet**(Se p. 9 bil. nr 2 och [p. 2:1 i Vägledning till ansökan](http://www.epn.se/media/43358/v_gledning_till_ans_kan_p2_1.doc))

Beskrivningen ska kunna förstås av nämndens samtliga ledamöter. Undvik därför terminologi som kräver specialkunskaper. Ange bakgrund och syfte för studien samt den/de vetenskapliga frågeställning (ar) som man söker svar på. Ange de viktigaste undersökningsvariablerna. Beskriv vilka kunskapsvinster projektet kan förväntas ge och betydelsen av dessa. Ange om det är en registerstudie, uppdragsforskning etc. För fackmän avsedd detaljerad information om forskningsplan/protokoll (program) *ska* bifogas som bilaga (se p. 9 bil. nr 2). För utformning av forskningsplan/protokoll se p. 2:1 i Vägledning till ansökan. Ange när datainsamlingen beräknas vara avslutad. En utförligare beskrivning av studiens genomförande *avsedd för lekmän* kan vid behov bifogas den för fackmän avsedda obligatoriska forskningsplanen.

Många personer äter mellanmål i syfte att döva hungern mellan huvudmåltiderna. Ofta föreslås att man äter frukt som mellanmål för att få i sig t.ex. vattenlösliga vitaminer, det finns nu flera företag som säljer just fruktkorgar vilka sponsras av arbetsgivare så att arbetstagaren kan få en frukt om dagen. Nyare data talar för att man skall begränsa sockerintaget för att minska fetmaepidemin, men denna debatt rör huvudsakligen föda som innehåller tillsatt socker. Flera moderna studier talar för att just fruktsocker, fruktos, som förekommer i frukt, innebär en ökad risk för metabola bieffekter inklusive minskad känslighet för det i kroppen producerade insulinet som har till uppgift att låta sockret komma in i cellerna från blodet (så kallad insulinresistens) och dessutom misstänks sockerarten fruktos orsaka fettlever, enligt en del omdebatterade studier. Än så länge har intresset för att studera det mer specifika effekterna av söta frukter, som man intar utan att först göra juice, varit mycket begränsat. Man kan tänka sig att fiberinnehållet i frukten som ätes, tuggas, som hel frukt utgör ett skydd mot metabola bieffekter av sådant intag, men det finns inte specifikt studerat enligt vad vi kan finna i vetenskaplig litteratur, i någon lottad studie där också leverförfettning undersökes.

Med så kallad leverspektroskopi kan mängden fett i levern fastställas utifrån strålningsfri magnetresonanstomografi (MR) teknik. Vi har använt denna teknik i tidigare undersökningar med gott resultat i studier på människa. Vi önskar nu göra en lottad studie under 2 månader där deltagare lottas till mellanmål i form av frukt eller till motsvarande extra kaloriintag från en källa med mindre fruktos, i form av nötter. Studien syftar fr.a. till att undersöka metabola effekter av dessa två kosttillskott på riskfaktorer för diabetes och hjärtkärlsjukdom med hjälp av blodprover, mätning av ämnesomsättning samt leverspektroskopi för bestämning av mängden fett i levern. Enkäter kommer också användas för att se om vi får effekter på livskvalitet och vi planerar också undersöka om tandstatus påverkas av de olika mellanmålen.

Studieupplägg

Rekrytering av ca 28 män och kvinnor som lottas till 7 kCal/kg kroppsvikt intag av nötter eller frukt som mellanmål.

Frukten får väljas och inköpas av deltagarna själva för att öka följsamheten. Kostnadsersättning mot kvitto i efterhand. Dagbok över fruktintag föres av deltagarna. Vi kommer i första hand önska att man konsumerar vanligt förekommande frukt såsom bananer, äpplen och päron (studien görs på hösten), gärna ekologist odlade frukter.

Nötsort får väljas och inköpas av deltagarna själva för att öka följsamhet. Kostnadsersättning mot kvitto i efterhand. Dagbok över nötintag föres av deltagarna. Vi kommer i första hand önska att man konsumerar vanligt förekommande nötter såsom valnötter, hasselnötter etc. Jordnötter räknas i detta fall också som en nöt (trots att det är en baljväxt egentligen).

Innan studien startas tas sjukhistoria, anamnes och status, och allmän rutinprovtagning (blodvärde, levervärden, njurar, ämnesomsättning etc) för att se så att ingen allvarlig sjukdom föreligger som kan påverka studieanalyserna (i så fall kan man inte vara med). Studenter i beroendeställning kan inte rekryteras som deltagare. Prover på ämnesomsättning samt rutinprover upprepas efter 8 veckor (avslut). Basmetabolism, så kallad indirekt kalorimetri, med registrering av koldioxidproduktion samt syrgasupptag utföres därtill i vila vid dessa tidpunkter samt magnetresonanstomografi för bestämning av leverfettmängd samt dessutom kroppssammansättning (muskler och fettfördelning). Den separata individuella analysen av MR bilderna sker blindat så långt detta är tekniskt möjligt. Tandstatus utföres av tandläkare före och efter perioden, i syfte att fr.a. klassa graden av eventuell tandstens och tandköttsinflammation (inte röntgen).

Undersökningar och prover före och efter (i slutet av perioden) lottning till nötter eller frukt som mellanmål:

Blodprover i fastande:Levervärden, blodvärde, elstatus, inflammationsprover, halt av c-vitamin i blodet, blodfetter, insulin, glukos, urat,

MR, leverspektroskopi

Indirekt kalorimetri

Kostregistrering under 3 dagar

Tandstatus (tandfickor)

Enkäter (SF 36)

Mätning av fysisk aktivitet (bära en accelerometer under 3 dagar)

Antropometri (längd, vikt, midjeomfång, blodtryck mm).

Deltagarna får ta del av prover och undersökningar efter studien och får också 2000 kr i ersättning (efter skatteavdrag) för att vara med

**2:2 Vilken/vilka vetenskaplig (a) frågeställning (ar) ligger till grund för projektets utformning?**

Om projektet kan karakteriseras som en hypotesprövning, ange den primära och eventuellt sekundära hypotesen. Hänvisning till mer detaljerad information för fackmän kan ske till bifogad forskningsplan/protokoll enligt punkt 2:1.

Ger ett ökat intag av frukt som mellanmål mindre fördelaktige effekter på riskfaktorer för kärlsjukdom och diabetes (blodfetter, insulin, leverfett mm) än motsvarande extra kalorimängd från nötter (som innehåller fett och protein snarare än olika sockerarter och vitaminer som i frukt)?.

**2:3 Redogör för resultat från relevanta djurförsök (Gäller klinisk behandlingsforskning)**

Om djurförsök inte utförts ange skälen till detta.

För att besvara den specifika effekten av mellanmål på riskfaktorer på människor i vardagsmiljö, kan inte djurförsök användas. Djur har andra levnadsbetingelser och en annan metabolism och ämnesomsättning.

**2:4 Redogör översiktligt för undersökningsprocedur, datainsamling och datas karaktär** ([Se p. 9 bil. nr 5 och p. 2:4 i Vägledning till ansökan](http://www.epn.se/media/43361/v_gledning_till_ans_kan_p2_4.doc))

Av beskrivningen ska framgå hur projektet planeras genomföras. Beskriv insamlade datas karaktär. Ange hur datas tillförlitlighet säkerställs (t.ex. kvalitetskontroll/monitorering). Vid enkäter och intervjuer ska beskrivas tillvägagångssätt och t.ex. frågors innehåll och hur slutsatser dras. Enkäter och skattningsskalor *ska* bifogas (se p. 9 bil. nr 5). För medicinsk forskning ska anges t.ex. typer av ingrepp, mätmetoder, antal besök, tidsåtgång vid varje försök, doser och administrationssätt för eventuella läkemedel och/eller isotoper, blodprovsmängd (även ackumulerad mängd vid multipla försök). Ange om och på vilket sätt undersökningsprocedur m.m. skiljer sig från klinisk rutin. Om en behandling studeras för första gången på människa ska detta framgå och relevanta säkerhetsrutiner beskrivas. Ange proceduren för att ge den eventuella behandling efter projektets slut, som kan erfordras. Ange procedur för insamling av biologiskt material. Redogör för datakällor och procedurer vid behandling av personuppgifter. För mer detaljerad information kan hänvisning ske till bilagd forskningsplan.

Undersökningar och prover före och efter (i slutet av perioden) lottning till nötter eller frukt som mellanmål:

Blodprover i fastande:Levervärden, blodvärde, elstatus, inflammationsprover, halt av c-vitamin i blodet, blodfetter, insulin, glukos, urat,

MR, leverspektroskopi

Indirekt kalorimetri

Kostregistrering under 3 dagar

Tandstatus

Enkäter (SF 36)

Mätning av fysisk aktivitet (accelerometer under 3 dagar)

Antropometri (längd, vikt, midjeomfång, blodtryck mm).

**2:5 Redogör för om insamlat biologiskt material kommer att förvaras i en biobank**

([Se p. 2:5 i Vägledning till ansökan](http://www.epn.se/media/43364/v_gledning_till_ans_kan_p2_5.doc))

*Med biobank avses biologiskt material från en eller flera människor som samlas och bevaras tills vidare eller för en bestämd tid och vars ursprung kan härledas till den eller de människor från vilka materialet härrör.*Redogör för var och hur prover som ska sparas förvaras, kodningsprocedurer och villkor för utlämnande av prover. Ange huvudman för biobanken.

Extra provrör för analys av metabolismrelaterade hormoner sparas nedfrysta, från undersökningarna före och efter frukt eller nötter, för analys av en del metabolismrelaterade hormoner, under några månader (då det kommer krävas att vi skickar en del av dessa för analys då det inte är rutinprover). Vi kommer inte spara provrör en längre tid. Alla data från detta kodas och kodlista hanteras av Fredrik Nyström

**2:6 Dokumentation, dataskydd och arkivering** ([Se p. 2:6 i Vägledning till ansökan](http://www.epn.se/media/43367/v_gledning_till_ans_kan_p2_6.doc))

Redogör för hur undersökningsprocedurer och eventuella ingrepp dokumenteras. Ange om band- och videoinspelningar används. Om materialet ska kodas, ange proceduren, vem som förvarar kodlistor/kodnycklar och vem eller vilka som har tillgång till dem, var och hur länge de förvaras samt om materialet kommer att anonymiseras eller förstöras. Redogör för vilken tillgänglighet datamaterialet har och hur det förvaras samt hur erforderligt sekretesskydd erhålls.

Kodlista för sammankoppling av avidentifierade data med faktiska personuppgifter/personnummer förs av huvudansvarig Fredrik Nyström. Inga ingrepp eller medicinska åtgärder utförs, och därmed sker ingen medicinsk journalföring i studien. Materialet kommer i original att sparas på Universitetssjukhuset. Avidentifierade datafiler kommer att hanteras inom universitetet (HU). Om avvikande prover som inger misstanke om sjukdom upptäcks hanteras detta på vanligt kliniskt vis och det hela handläggs på Endokrinmedicin med sedvanlig journalföring och utredning utanför studien där Fredrik Nyström är huvudansvarig

**2:7 Redogör för tidigare erfarenheter (egna och/eller andras) av den använda
proceduren, tekniken eller behandlingen**

Särskilt angeläget är att redovisning av risker för komplikationer görs tydliga och i förekommande fall med angivande av relevanta publikationer. Vid nya behandlingar av patienter, t.ex. med läkemedel, bör anges hur många patienter (med aktuell eller annan åkomma) som tidigare erhållit föreslagen behandling, läkemedelsdosering (eller annan dosering) samt hur långa behandlingsperioder som studerats.

Vi har tidigare använt samtliga metoder på friska frivilliga tidigare. Undersökningarna är inte riskabla eller smärtsamma, annat än möjligen risken för blåmärken vid venprovtagning.

**3. Uppgifter om forskningspersoner**

**3:1 Hur görs urvalet av forskningspersoner?** ([Se p. 9 bil. nr 3 och p. 3:1 i Vägledning till ansökan](http://www.epn.se/media/43370/v_gledning_till_ans_kan_p3_1.doc))

*Med forskningsperson avses en levande människa som forskningen avser.* Ange urvalskriterier (inklusion och exklusion). Redogör för på vilket sätt forskaren kommer i kontakt med/får kännedom om lämpliga forskningspersoner. Om annonsering sker, *ska* annonsmaterialet insändas som bilaga (se p. 9 bil. nr 3). Om t.ex. barn eller personer som tillfälligt eller permanent inte är kapabla att ge ett eget informerat samtycke ska ingå i projektet, ska detta särskilt motiveras. Om vissa grupper utesluts från deltagande i projektet ska detta särskilt motiveras.

Vi kommer inkludera män och kvinnor över 18 år som inte har allvarliga sjukdomar som gör provtagningen och analyserna otillförlitliga. Leversjukdom utgör ett eklusionskriterium (rutinblodprover tas för att kontrollera detta). Psykisk sjukdom och svårigheter att förstå instruktioner innebär också exklusionskriterier.

**3:2 Ange relationen mellan forskare/försöksledare och forskningspersonerna**

Behandlare (t.ex. läkare, psykolog, sjukgymnast) - forskningsperson (t.ex. patient, klient)

Kursgivare (lärare) - student

Arbetsgivare - anställd

Annan relation som kan tänkas medföra risk för påverkan. Beskriv: forskningsledare-frivillig frisk försöksperson

3:3 Redogör för det statistiska underlaget för studiepopulationens (-ernas)/ undersökningsmaterialets (-ens) storlek [**(Se p. 3:3 i Vägledning till ansökan)**](http://www.epn.se/media/43373/v_gledning_till_ans_kan_p3_3.doc)

Redovisa statistisk styrka, så kallad ”power”- beräkning eller redovisa motsvarande överväganden som tydliggör studiens möjligheter att besvara frågeställningarna.

Baserat på våra tidigare jämförande studier av olika kostinterventioner så har 28 personer varit tillräckligt för att påvisa kliniskt relevanta skillnader i leverförfettning (och på metabola riskmarkörer), det ger oss 80% power att se en ökning på 50% av frukt på leverförfettning, se forskningsprogram.

**3:4 Kan forskningspersonerna komma att inkluderas i flera studier samtidigt
eller i nära anslutning till denna? I så fall, vilken typ av forskning?**

([Se p. 3:4 i Vägledning till ansökan](http://www.epn.se/media/43376/v_gledning_till_ans_kan_p3_4.doc))

Nej

3:5 Vilket försäkringsskydd finns för de forskningspersoner som deltar i projektet?

Det åligger forskningshuvudmannen att kontrollera att det finns försäkring som täcker eventuella skador som kan uppkomma i samband med forskningen.

Vi kan inte se att undersökningarna innnebär några reella risker, men patientförsäkringen gäller för komplikationer till t.ex. venprovtagningen.

**3:6 Vilken ekonomisk ersättning eller andra förmåner utgår till de forskningspersoner
som deltar i projektet och när betalas ersättningen ut?** Utförligare beskrivning kan lämnas i bilaga. ([Se p. 9 bil. nr 11 och p. 3:6 i Vägledning till ansökan](http://www.epn.se/media/43379/v_gledning_till_ans_kan_p3_6.doc))

Ersättning för obehag och besvär. Ange belopp (före skatt): 2000 kr efter skatt

Ersättning för förlorad arbetsinkomst  Ja  Nej

Reseersättning  Ja  Nej

Befrielse från kostnader för läkemedel  Ja  Nej

Befrielse från andra kostnader. Vilka?

Andra förmåner. Vilka?

När betalas ersättningen ut? Efter genomförd studie

Ingen ersättning betalas ut

### 4. Information och samtycke

4:1 Proceduren för och innehållet i den *information* som lämnas då forskningspersoner tillfrågas om deltagande

(Se p. 9 bil. nr 4 och [**Vägledning till forskningspersonsinformation**](http://www.epn.se/media/45467/v_gledning_till_forskningspersonsinformation_2012-03-27.doc).)

Enligt 16 § lag (2003:460) om etikprövning av forskning som avser människor ska forskningspersonen informeras om den övergripande planen för forskningen, syftet med forskningen, de metoder som kommer att användas, de följder och risker som forskningen kan medföra, vem som är forskningshuvudman, att deltagande i forskningen är frivilligt och forskningspersonernas rätt att när som helst avbryta sin medverkan. Beskriv hur och när information ges och vad den innehåller. Ange vem som informerar. Normalt ska en kortfattad och lättförståelig skriftlig information ges. Denna skriftliga information *ska* bifogas ansökan (se p. 9 bil. nr 4). Om ingen eller ofullständig information ges, måste skälen för detta noggrant anges.

Försökspersonerna informeras såväl muntligt som skriftligt (se bilaga) av den studierepresentant som träffar dem i rekryteringssyfte

**4:2 Hur och från vem inhämtas *samtycke*?** (Se [Vägledning till forskningspersonsinformation](http://www.epn.se/media/45467/v_gledning_till_forskningspersonsinformation_2012-03-27.doc))

Beskriv proceduren; vem som frågar, när detta sker och hur samtycket dokumenteras. Utförlig redovisning är särskilt viktig då barn eller personer med nedsatt beslutskompetens ingår i studien, likaså vid studier av en grupp/grupper, t.ex. skolklasser, föreningar, organisationer, företag, kyrkosamfund, församlingar eller grupper som interagerar inom sociala medier.

Försökspersonerna informeras såväl muntligt som skriftligt av den studierepresentant som träffar dem i rekryteringssyfte. Dokumentation genom skriftligt samtycke, se bilaga

### 5. Forskningsetiska överväganden

5:1 Redogör för alla risker som deltagandet kan medföra

Dessa kan vara t.ex. fysisk eller psykisk skada, smärta, obehag eller integritetsintrång på kort eller lång sikt. Ange vilka åtgärder som har vidtagits för att förebygga riskerna som nämns ovan samt vilken beredskap som finns för att hantera sådana komplikationer. Ange vilka/de metoder som kommer att användas för att efterforska, registrera och rapportera oönskade händelser.

Deltagarna utsätts för minimala risker, endast provtagningen bedöms kunna ge upphov till t.ex. blåmärken. Vi kommer bara rekrytera deltagare som accepterar och är helt införstådda med detta och vi bedömer också att det är väsentligen ofarligt att äta olika former av mellanmål under 8 veckor

5:2 Redogör för möjlig nytta för de forskningspersoner som ingår i projektet (gäller särskilt behandlingsforskning)

Erfarenhet av att deltaga i ett forskningsprojekt, MR av kroppen samt information om ämnesomsättning, blodprover mm

**5:3 Identifiera och precisera om eventuella etiska problem (fördelar/nackdelar) kan uppstå i ett vidare perspektiv genom projektet** (Se [p. 5:3 i Vägledning till ansökan](http://www.epn.se/media/43382/v_gledning_till_ans_kan_p5_3.doc))

Här kan redovisas om exempelvis vissa grupper (andra än de forskningspersoner som ingår i forskningsprojektet) kan komma att utpekas/få hjälp som ett resultat av studien.

Om vi finner att frukt är mindre fördelaktigt och att det ökar risken för leverförfettning eller tandköttsinflammation så kan detta leda till debatt om detta verkligen är lämpligt som mellanmål. I så fall kan man tänka sig att åtminstone en del hälsoråd kan komma att justeras och inte rekommendera intag av söta frukter. Men detta är beroende på studiens utfall och på hur tydliga resultaten blir.

### 6. Redovisning av resultaten

**6:1 Hur garanteras forskningshuvudmannen och medverkande forskare tillgång till data (anges vid t.ex. uppdragsforskning) och vem ansvarar för databearbetning och rapportskrivning?**([Se p. 6:1 i Vägledning till ansökan](http://www.epn.se/media/43385/v_gledning_till_ans_kan_p6_1.doc))

Fredrik Nyström ansvarar för datainsamling samt vetenskaplig rapport i referee-granskad internationell tidskrift

6:2 Hur kommer resultaten att göras offentligt tillgängliga? Kommer studien att insändas för publicering i tidskrift eller publiceras på annat sätt? ([**Se p. 6:2 i Vägledning till ansökan**](http://www.epn.se/media/43388/v_gledning_till_ans_kan_p6_2.doc))

Ange i vilken form resultaten planeras offentliggöras samt tidsplan för detta.

Ja, rapport i referee-granskad internationell tidskrift

6:3 På vilket sätt garanteras forskningspersonernas rätt till integritet när materialet offentliggörs/publiceras?

Redovisas resultat på statistisk gruppnivå? Beskriv procedurer eller metoder för avidentifiering/anonymisering.

Resultaten presenteras på gruppnivå. All presentation är helt avidentifierad

7. Redovisning av ekonomiska förhållanden och beroendeförhållanden

Redovisning enligt punkterna 7:1-7:3 syftar till att tydliggöra alla direkta eller indirekta förhållanden, som kan tänkas påverka forskarens relation till forskningspersonerna (vid t.ex. informations-, samtyckes-, genomförandeprocedurer).

7:1 Vid uppdragsforskning

Ange uppdragsgivaren t.ex. ett företag (vid klinisk läkemedelsprövning eller prövning av andra nya produkter), en organisation eller en myndighet.

Namn: Ej aktuellt Kontaktperson:

Adress:       Telefon/mobiltelefon:

Ange uppdragsgivarens relation till forskningshuvudmannen/medverkande forskare, t.ex.
anställningsförhållande

Ej aktuellt

**7:2 Redovisa eventuella ekonomiska överenskommelser med uppdragsgivare eller**

**andra finansiärer (namn, belopp)**

Vid klinisk läkemedelsprövning bör hänvisning ske till ingånget avtal med sjukvårdshuvudmannen. Liknande överenskommelser kan förekomma vid annan uppdragsforskning och bör redovisas på samma sätt. Separata överenskommelser med den/de som ska genomföra forskningen ska redovisas. Belopp som kommer att erhållas för studien/ersättning till kliniken/genomföraren, vad ersättningen bör täcka och ev. belopp som erhålls per forskningsperson, bör också anges här.

Ej aktuellt

7:3 Redovisa forskningshuvudmannens, huvudansvarig forskares och medverkande forskares egna intressen

Här redovisas t.ex. aktieinnehav, anställning, konsultuppdrag i finansierande företag, eget företag som kan få (direkt eller indirekt) ekonomisk vinst av forskningen.

Inga särintressen föreligger mer än intresset att undersöka relevansen av vanliga kostråd

**8. Undertecknande**

Behörig företrädare för sökande forskningshuvudman enligt p. 1:2.

Ort:       Datum:

Signatur: __________________________________________________________________

Namnförtydligande:

Tjänstetitel:

Undertecknad forskare som genomför projektet (kontaktperson) enligt p. 1:3 intygar härmed att forskningen kommer att genomföras i enlighet med ansökan.

Ort:       Datum:

Signatur: _________________________________________________________________

Namnförtydligande:

Tjänstetitel:

**9. Förteckning över bilagor** ([Se p. 9 i Vägledning till ansökan](http://www.epn.se/media/43391/v_gledning_till_ans_kan_p9.doc))

Dokument som, i tillämpliga fall, ska bifogas *om inte motsvarande information finns i blanketten* har markerats med x. Markera de bilagor som skickas in med denna ansökan.

| **Insänd med ansökan** | **Bil nr** | Beskrivning | **Klinisk läkemedels-**  **prövning** | **Annan forskning** |
| --- | --- | --- | --- | --- |
|  | 1 | Deltagande forskningshuvudmän och medverkande forskare (kontaktpersoner) vid forskning där mer än en forskningshuvudman deltar. Se p. 1:4 | x | x |
|  | 2 | För fackmän avsedd forskningsplan, vid behov även för lekmän avsedd bilaga. Se p. 2:1 och Vägledning till forskningsplan/forskningsprotokoll (program) | x | x |
|  | 3 | Annonsmaterial för rekrytering av forskningspersoner. Se p. 3:1 och i Vägledning till ansökan p. 3:1 | x | x |
|  | 4 | Skriftlig information till dem som tillfrågas. Se p. 4:1 och Vägledning till forskningspersonsinformation och (i förekommande fall) separat samtyckesformulär | x | x |
|  | 5 | Enkät, frågeformulär. Se p. 2:4 | x | x |
|  | 6 | Gemensam EU blankett (gäller fr.o.m. den 1 maj 2004), gäller även vid ändring. För information se Läkemedelsverkets hemsida, [www.lakemedelsverket.se](http://www.lakemedelsverket.se/) | x |  |
|  | 7 | Sammanfattning av protokollet på svenska | x |  |
|  | 8 | Prövarhandbok alt. bipacksedel/produktresumé/IB | x |  |
|  | 9 | Intyg från verksamhetschef/motsv. om resurser för forskningspersonernas säkerhet. Se p. 1:5 och förslag till utformning av resursintyg i Vägledning till ansökan p. 1:5 | x | x |
|  | 10 | CV för forskare (samma som p. 1:3) med huvudansvar för genomförandet, redovisa forskarens (- arnas) kompetens av relevans för studien. Se Vägledning till ansökan p. 1:3 | x | x |
|  | 11 | Beskrivning av ersättning till forskningspersoner. Se p. 3:6 och  i Vägledning till ansökan p. 3:6 | x | x |

**Övriga bilagor som bifogas ansökan:**
